# Discovery of Metal Ions Chelator Quercetin Derivatives with Potent Anti-HCV Activities

**DOI:** 10.3390/molecules20046978

**Published:** 2015-04-16

**Authors:** Dongwei Zhong, Mingming Liu, Yang Cao, Yelin Zhu, Shihui Bian, Jiayi Zhou, Fengjie Wu, Kum-Chol Ryu, Lu Zhou, Deyong Ye

**Affiliations:** 1 Department of Medicinal Chemistry, School of Pharmacy, Fudan University, 826 Zhang-Heng Rd, Shanghai 201203, China; E-Mails: 12211030012@fudan.edu.cn (D.Z.); 09111030009@fudan.edu.cn (M.L.); 14111030010@fudan.edu.cn (Y.C.); 09301030045@fudan.edu.cn (Y.Z.); 08301030057@fudan.edu.cn (S.B.); 09301030009@fudan.edu.cn (J.Z.); 11307120169@fudan.edu.cn (F.W.); 2 Institute of Pharmacy, HamHung Pharmaceutical University, HamHung 999093, Democratic People’s Republic of Korea; E-Mail: yegroup@126.com

**Keywords:** anti-HCV activities, diketoacids mimic, quercetin derivatives, synthesis, docking

## Abstract

Analogues or isosteres of α,γ-diketoacid (DKA) **1a** show potent inhibition of hepatitis C virus (HCV) NS5B polymerase through chelation of the two magnesium ions at the active site. The anti-HCV activity of the flavonoid quercetin (**2**) could partly be attributed to it being a structural mimic of DKAs. In order to delineate the structural features required for the inhibitory effect and improve the anti-HCV potency, two novel types of quercetin analogues, 7-*O*-arylmethylquercetins and quercetin-3-*O*-benzoic acid esters, were designed, synthesized and evaluated for their anti-HCV properties in cell-based assays. Among the 38 newly synthesized compounds, 7-*O*-substituted derivative **3i** and 3-*O*-substituted derivative **4f** were found to be the most active in the corresponding series (EC_50_ = 3.8 μM and 9.0 μΜ, respectively). Docking studies suggested that the quercetin analogues are capable of establishing key coordination with the two magnesium ions as well as interactions with residues at the active site of HCV NS5B.

## 1. Introduction

It was estimated that about 170 million people worldwide were chronically infected by hepatitis C virus (HCV) and were consequently at risk of developing liver cirrhosis and/or hepatocellular carcinoma [1]. A protective vaccine is not yet available and the previous standard of care (SOC), which is pegylated-interferon, combined with ribavirin, is often difficult to tolerate and results in a sustained viral response (SVR) in only 50% of patients infected with the predominant genotype 1 [2,3]. Recent advances in the development of direct acting antivirals (DAAs) have significantly improved SVR in patients, providing a new hope for cure in infected patients. Three NS3/4A protease inhibitors were approved by FDA for the treatment of genotype 1 hepatitis C, and in late 2013, sofosbuvir, a first-in-class NS5B polymerase inhibitor, was launched and became a cornerstone of recommended HCV therapy against almost all HCV genotypes [4,5,6,7]. However, due to the potential of drug resistant strains [8] and the high price of the new HCV drugs [9], the development of novel anti-HCV agents is still an urgent necessity.

The RNA-dependent RNA polymerase (RdRp) of HCV, also known as protein NS5B, is a key enzyme in the synthesis of a complementary minus-strand RNA and the subsequent synthesis of a genomic plus-strand RNA from this minus-strand RNA template [10]. In the enzymatic reactions, two divalent cations in the active site, such as magnesium (Mg^2+^) or manganese (Mn^2+^) play a critical role in the ligation of the ribonucleotide triphosphate (rNTP) substrates and the promotion of a favorable geometry of the active site. The pivotal role of the divalent cations in the active site of the HCV polymerase supports the rational basis of the inhibition of this enzyme by metal chelating motifs.

**Figure 1:**
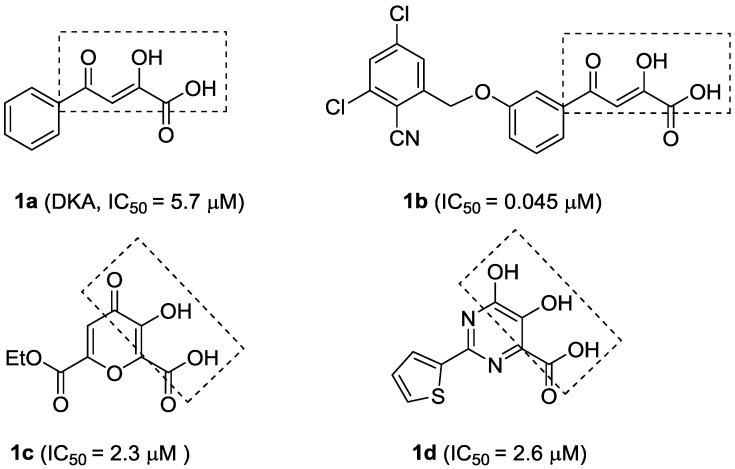
Structures and activities of α,γ-diketoacids (DKAs) and their analogues or isosteric derivatives.

The α,γ-diketoacid (DKA) **1a** (Figure 1) has been identified as a selective and reversible inhibitor against NS5B through high-throughput screening approaches. Subsequent structure-activity relationship (SAR) optimization led to **1b** (Figure 1), a potent HCV polymerase inhibitor [11]. The mode of action of DKAs involves chelation of the metal ions present in the active site of the HCV RdRp [12]. However, due to their unfavorable physicochemical and pharmacokinetic properties [13], many DKA analogues or isosteres with NS5B inhibitory activities, including meconic acid [14] (**1c**, Figure 1) and dihydroxypyrimidine carboxylic acid [12] (**1d**, Figure 1), have been designed and prepared, by keeping the metal-binding moieties to achieve effective chelation of the two Mg^2+^ ions.

**Figure 2:**
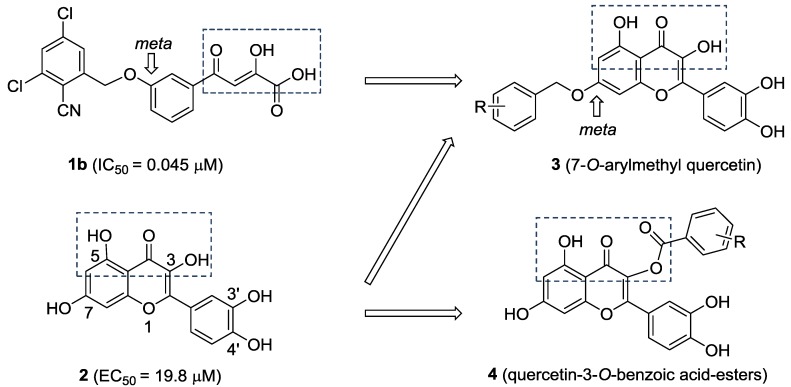
The design of 7-*O*-arylmethylquercetin derivatives **3** and quercetin-3-*O*-benzoic acid ester derivatives **4**.

Quercetin (**2**, Figure 2) is a naturally occurring flavonoid with various biological activities such as antioxidant, antiviral, anticancer, antimicrobial and anti-inflammatory properties; hence, quercetin and its derivatives have potential as drug development leads [15]. As a part of our studies on anti-HCV agents, quercetin was identified as an HCV inhibitor with moderate antiviral activity (EC_50_ = 19.8 μM) [16]. Furthermore, it had been reported that quercetin could inhibit HCV RdRp at low micromolar concentration (83.7% inhibition at the concentration of 20 μM in an enzyme assay) [17]. We reasoned that the adjacent carbonyl and hydroxyl groups on A and C rings of quercetin make it a perfect DKAs mimic to chelate the two metal ions. In consideration of the structures of **1a** and **1b** (Figure 1), introduction of a 2-cyano benzyl ether group at the aromatic *meta*-position relative to the chelating moieties was shown to significantly improve the antiviral activity. Thus, we anticipated that substituents of quercetin at the 7-*O* position (7-*O*-arylmethyl quercetins **3**, Figure 2) would mimic the *meta*-substituted DKA **1b** and enhance the anti-HCV activities. Herein, we report the design and synthesis of novel 7-*O*-arylmethyl quercetin derivatives with various aromatic substituents and the evaluation of their anti-HCV activities. Moreover, it can be hypothesized that another introduced carbonyl group at 3-position of quercetin, together with the adjacent carbonyl and hydroxyl group, would be capable of chelating the two metal ions. Hence, this paper also described a series of novel quercetin-3-*O*-benzoic acid esters (compounds **4**, Figure 2) in an attempt to investigate whether the introduction of another carbonyl group at 3-position would result in an improved anti-HCV activity. In addition, several quercetin derivatives with different aliphatic groups substituted at the 3-position were also synthesized for further SAR investigations.

## 2. Results and Discussion

### 2.1. Chemistry

**Scheme 1:**
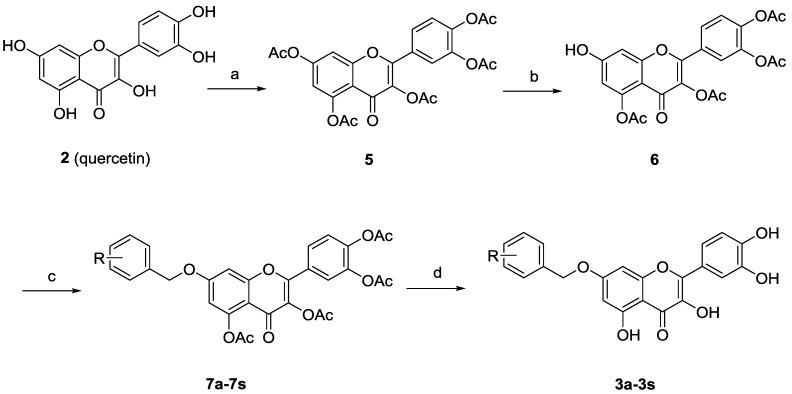
Synthesis of the 7-*O*-substituted quercetin derivatives.

Synthesis of 7-*O*-substituted quercetin derivatives is well documented [18]. As depicted in Scheme 1, peracetylation of quercetin (**2**) followed by regioselective deacetylation of 7-OAc group with thiophenol and imidazole in *N*-methyl-2-pyrrolidone (NMP) at 0 °C gave the 7-*O*-monodeprotected flavonol **6**. Alkylation of **6** with variously substituted benzyl bromides, followed by deacetylation by treatment with methanolic ammonia provided the desired 7-*O*-arylmethylquercetins **3a**–**3s**.

**Table:** 

	R		R		R
**3a**	H				
**3b**	*o*-F	**3h**	*m*-F	**3n**	*p*-F
**3c**	*o*-Cl	**3i**	*m*-Cl	**3o**	*p*-Cl
**3d**	*o*-Br	**3j**	*m*-Br	**3p**	*p*-Br
**3e**	*o*-CH_3_	**3k**	*m*-CH_3_	**3q**	*p*-CH_3_
**3f**	*o*-CN	**3l**	*m*-CN	**3r**	*p*-CN
**3g**	*o*-NO_2_	**3m**	*m*-NO_2_	**3s**	*p*-NO_2_

*Reagents and conditions:* (**a**) Ac_2_O, pyridine, reflux, 6 h, 79%; (**b**) PhSH, imidazole, NMP, 0 °C, 1 h, 70%; (**c**) R-PhCH_2_Br, K_2_CO_3_, acetone, rt, 3 h; (**d**) 2M NH_3_/MeOH, 0 °C, 1 h, 40%–56% (two steps).

**Scheme 2:**
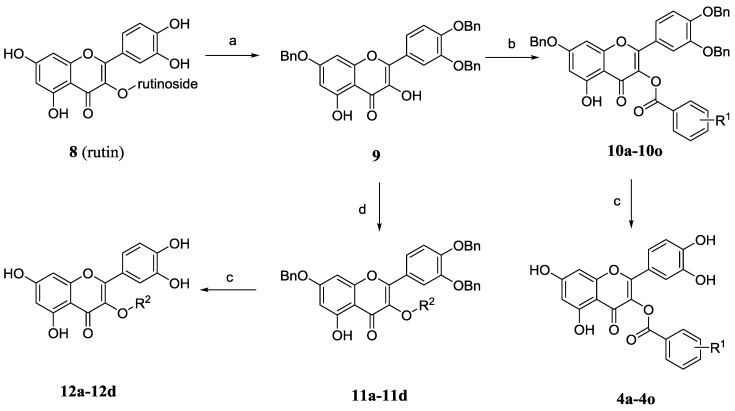
Synthesis of the quercetin-3-*O*-substituted benzoic acid esters and 3-*O*-substituted quercetin derivatives.

The synthesis of 3-*O*-substituted quercetin derivatives is outlined in Scheme 2. In the light of the synthetic route in the literature [19,20], tribenzylation of rutin (**8**) with potassium carbonate and benzyl bromide in DMF followed by removal of the *C*3-rutinose with hydrochloric acid and ethanol gave 3',4',7-tri-*O*-benzylquercetin (**9**). Compound **9** was condensed with variously substituted benzoic acids, 1-ethyl-3-(3-dimethylaminopropyl) carbodiimide (EDCI) and a catalytic amount of 4-dimethyl-amiopryidine (DMAP) to afford the relevant quercetin-3-*O*-ester derivatives **10a**–**10o**. Alternatively, the etherification of compound **9** was performed in the presence of potassium carbonate with diverse bromides or iodides to afford relevant quercetin-3-*O*-ether derivatives **11a**–**11d**. Finally, the desired final products quercetin derivatives **4a**–**4o** and **12a**–**12d** were obtained by hydrogenolysis and further purification by silica gel chromatography.

**Table:** 

	R^1^		R^1^		R^1^		R^2^
**4a**	H	**4f**	3''-F	**4k**	4''-F	**12a**	Me
**4b**	2''-F	**4g**	3''-NH_2_	**4l**	4''-NH_2_	**12b**	*i*-Pr
**4c**	2''-NH_2_	**4h**	3''-OCH_3_	**4m**	4''-OCH_3_	**12c**	(CH_2_)_2_OH
**4d**	2''-OCH_3_	**4i**	3''-CN	**4n**	4''-CN	**12d**	(CH_2_)_3_CN
**4e**	2''-CN	**4j**	3''-Cl	**4o**	4''-Cl		

*Reagents and conditions*: (**a**) K_2_CO_3_, BnBr, DMF, 60 °C, 3 h, then HCl con., ethanol, 70 °C, 2 h, 85%; (**b**) R^1^-PhCOOH, EDCI, DMAP, DMF, rt, overnight; (**c**) H_2_, Pd/C, 1,4-dioxane/ethanol, rt, 6 h, 40%–79% (two steps); (**d**) R^2^Br or R^2^I, K_2_CO_3_, DMF, 40 °C, 3 h.

### 2.2. Biological Evaluation

The inhibition of HCV by all synthesized quercetin derivatives was evaluated following a previously reported procedure [16]. All newly synthesized compounds were tested in authentic HCV infection/replication system in the human hepatoma cell lines Huh7.5.1 using mycophenolic acid [21] (MPA) as control compound. The cytotoxicity effect of the test compounds was also evaluated in the same cell line by MTT method. Antiviral effect and cytotoxicity effect were summarized as EC_50_ and CC_50_ respectively, as well as the selective index (SI) which is the ratio of CC_50_ to EC_50_. The stability of quercetin-3-*O*-ester compounds were also tested in the same buffer as the antiviral activity assay at 37 °C and the hydrolysate was measured by HPLC. Only trace quercetin as hydrolyzate was monitored over 72 h which demonstrated that quercetin-3-*O*-ester derivatives were stable under the biological evaluation conditions.

As clearly shown in Table 1, all 7-*O*-substituted quercetin derivatives **3a**–**3s** exhibited potent anti-HCV activities with EC_50_ values ranging from 3.8 μM to 8.7 μM. Among these compounds, 3''-Cl substituted derivatives possessed the highest activities (for **3i**, EC_50_ = 3.8 μM) and a decent selectivity ratio (for **3i**, SI = 7.2). These results tend to indicate the importance of introducing an aromatic group at 7-*O* position. Substituents of quercetin at the 7-*O* position may mimic the *meta*-substituents of DKA **1b** and thus enhance the anti-HCV activities. It should be noted that all the 7-*O*-substituted analogues evaluated in this study were found in a narrow range of inhibition activity. This high level of tolerance will be discussed in the following section.

**Table 1:** Anti-HCV activities and cytotoxic effects of quercetin-7-*O*-substituted derivatives. 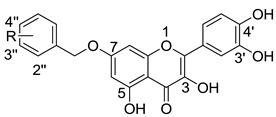

Compound	R		EC_50_(μM) ^a,b,c^	CC_50_(μM) ^c,d^	SI ^e^
	Position	Substituent			
**3a**	-	H	8.7 ± 0.2	>50	>5.8
**3b**	2''	F	5.0 ± 0.1	15.4 ± 0.1	3.1
**3c**		Cl	4.8 ± 0.1	36.7 ± 0.7	7.7
**3d**		Br	5.5 ± 0.2	37.4 ± 0.8	6.8
**3e**		CH_3_	4.9 ± 0.6	21.7 ± 1.4	4.4
**3f**		CN	7.5 ± 0.5	12.6 ± 0.1	1.7
**3g**		NO_2_	7.0 ± 1.1	14.6 ± 0.5	2.1
**3h**	3''	F	6.3 ± 0.2	44.5 ± 3.7	7.7
**3i**		Cl	3.8 ± 0.2	27.2 ± 0.2	7.2
**3j**		Br	4.1 ± 0.3	22.3 ± 1.2	5.4
**3k**		CH_3_	4.7 ± 0.4	32.5 ± 0.7	6.9
**3l**		CN	5.4 ± 0.5	10.6 ± 0.5	2.0
**3m**		NO_2_	6.6 ± 1.1	15.0 ± 0.1	2.3
**3n**	4''	F	5.1 ± 0.5	31.9 ± 3.4	6.2
**3o**		Cl	3.9 ± 0.3	15.1 ± 0.1	3.9
**3p**		Br	5.5 ± 1.5	35.4 ± 1.6	6.4
**3q**		CH_3_	6.7 ± 1.4	15.9 ± 1.0	2.4
**3r**		CN	6.2 ± 0.2	10.1 ± 0.1	1.6
**3s**		NO_2_	4.3 ± 0.2	19.6 ± 0.2	4.5
**2**	quercetin		19.8	>50	>2.5

^a^ The anti-HCV assay was evaluated in an authentic HCV infection system in the human hepatoma cell lines, Huh7.5.1; ^b^ Concentration required to inhibit HCV RNA replication by 50%; ^c^ The values obtained as the average of duplicate determinations; ^d^ Concentration required to reduce cell proliferation by 50%; ^e^ Selectivity index: ratio of CC_50_ to EC_50_.

To examine the effects of the additional carbonyl group at the 3-position, 3-*O*-substituted quercetin derivatives were designed by adding substituted aroyl groups. All the substituted benzoic acid esters **4a**–**4o** exhibited similar or better anti-HCV activities in cell-based assays as compared to the lead compound quercetin (Table 2).

**Table 2:** Anti-HCV activities and cytotoxic effects of quercetin-3-*O*-substituted derivatives. 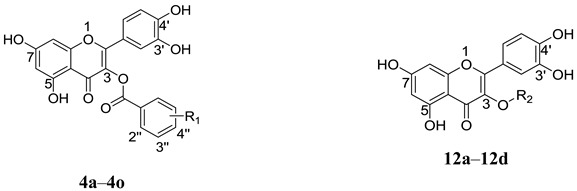

Compound	R^1^		R^2^	EC_50_(μM) ^a,b,c^	CC_50_(μM) ^c,d^	SI ^e^
	Position	Substituent				
**4a**	-	H	-	14.3 ± 5.1	>50	>3.5
**4b**	2''	F	-	12.9 ± 3.2	>50	>3.9
**4c**		NH_2_	-	12.8 ± 2.8	>50	>3.9
**4d**		OCH_3_	-	11.7 ± 1.3	>50	>4.3
**4e**		CN	-	20.2 ± 8.6	>50	>2.5
**4f**	3''	F	-	9.0 ± 4.5	>50	>5.6
**4g**		NH_2_	-	21.1 ± 1.3	>50	>2.4
**4h**		OCH_3_	-	15.6 ± 0.3	>50	>3.2
**4i**		CN	-	13.1 ± 1.0	>50	>3.8
**4j**		Cl	-	23.0 ± 3.2	>50	>3.5
**4k**	4''	F	-	9.7 ± 3.1	35.0 ± 2.4	3.6
**4l**		NH_2_	-	23.8 ± 0.8	>50	>2.1
**4m**		OCH_3_	-	13.6 ± 2.1	33.9 ± 3.7	2.5
**4n**		CN	-	11.4 ± 2.3	>50	>4.4
**4o**		Cl	-	14.3 ± 2.2	>50	>3.5
**12a**	-	-	Me	11.2 ± 0.1	>50	>4.5
**12b**	-	-	*i*-Pr	38.9 ± 2.5	28.8 ± 2.0	0.7
**12c**	-	-	(CH_2_)_2_OH	26.3 ± 3.1	>50	>1.9
**12d**	-	-	(CH_2_)_3_CN	>50	>50	NA
**2**	quercetin			19.8	>50	>2.5

^a^ The anti-HCV assay was evaluated in an authentic HCV infection system in the human hepatoma cell lines, Huh7.5.1; ^b^ Concentration required to inhibit HCV RNA replication by 50%; ^c^ The values obtained as the average of duplicate determinations; ^d^ Concentration required to reduce cell proliferation by 50%; ^e^ Selectivity index: ratio of CC_50_ to EC_50_.

Among these compounds, 3''-F substituted derivative (**4f**) showed more than a two-fold increase in potency over quercetin (EC_50_ of 9.0 μM for **4f** versus 19.7 μM for **2**). In order to study the significance of the coordination between the additional carbonyl group and the magnesium ions, we synthesized several compounds **12a**–**12d** in which the carbonyl group at 3-O position was replaced by aliphatic groups of various lengths. It is not surprising that a decrease in potency was observed for these analogues, mainly because the aliphatic substituents at the 3-position, especially when they are bulky, could not coordinate to the magnesium ions. The maintenance of anti-HCV activities for quercetin-3-*O*-benzoic acid-ester derivatives instead of the 3-*O*-aliphatic substituents revealed that the introduction of an electronegative carbonyl group at 3-*O* position could enhance the chelation ability between the inhibitor and metal ions, which contribute to the binding affinity in a similar way as the 3-hydroxyl group.

### 2.3. Molecular Docking

**Figure 3:**
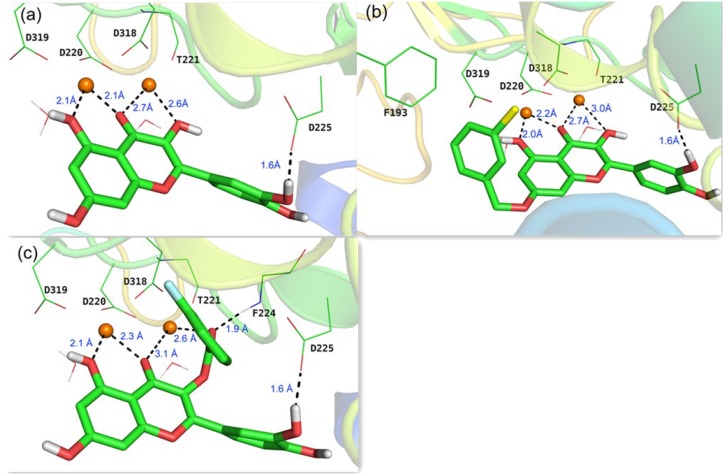
Predicted binding modes in the active site of a reported X-ray structure of HCV RdRp (PDB code: 1GX6 [22]) from docking studies: (**a**) quercetin **2**; (**b**) compound **3i**; (**c**) compound **4f**. The two Mg^2+^ ions are indicated as orange spheres, carbon atoms are shown in green, oxygen in red, chlorine in yellow, and fluorine in blue.

To rationalize the observed activities of different substituents of quercetin and investigate the binding modes with NS5B, docking calculations were performed on the basis of the reported X-ray crystallographic structure of HCV genotype 1b RdRp complexed with uridine triphosphate (UTP) and divalent metal ions (PDB code: 1GX6 [22]). Initially, the lead compound quercetin **2** was docked into NS5B active site after the removal of the original UTP. As depicted in Figure 3a, 3-, 4-, and 5-*O* derivatives of quercetin chelate to the two magnesium ions (2.1 Å–2.7 Å) together with four conserved key residues (Asp 220, Thr221, Asp318, Asp319) and two water molecules in the active site to form a double-octahedral center. The three acidic residues (Asp220, Asp318, Asp319) were found to be indispensable to the polymerase-catalyzed nucleotidyl transfer reaction [22]. The 3'-hydroxyl in B ring of quercetin forms a hydrogen bond (1.6 Å) with the side chain of Asp225, the same residue that is hydrogen bonded to the 2'-hydroxyl group of UTP. This binding mode is in accordance with our previous results [16] that kaempferol (lacking a 3'-hydroxyl as compared to quercetin, 51.9% inhibition at 50 μM) and isorhamnetin (with 3'-hydroxy methylated, 47.3% inhibition at 50 μM) are less potent against HCV than quercetin (EC_50_ = 19.8 μM).

Representatives of 7-*O*-substituted and 3-*O*-substituted quercetin derivatives (**3i** and **4f**, respectively) were also docked into the active site of NS5B. In addition to the similar chelation and hydrogen bond found in quercetin-NS5B system, the 7-*O* phenyl group of **3i** forms a hydrophobic interaction with Phe193 (Figure 3b). These findings could potentially account for the much better activities of 7-*O*-substituent compared to quercetin (Table 1). However, due to the relatively large size of the hydrophobic pocket around Phe193, modification of 7-*O* phenyl group did not give a noticeable gain of activity. As to the 3-*O*-substituted analogue **4f**, the carbonyl group at 3-position interacts with one magnesium ion with a distance of 2.6 Å, the same distance as that in quercetin-NS5B system (Figure 3a,c). Interestingly, an extra hydrogen bond (1.9 Å) was found between the carbonyl group at 3-position and the backbone NH of Phe224, which may contribute to the better activities of quercetin-3-*O*-substituted derivatives compared to quercetin (Table 1).

## 3. Experimental Section

### 3.1. General Procedures

All solvents and reagents were purchased from commercial sources and used directly without purification. Progress of all the reactions were monitored by thin-layer chromatography (TLC) with precoated silica gel plates GF_254_, 10–40 µm (Huanghai, Yantai, China). Melting points (m.p.) were determined using an X-4 microscope melting point apparatus and are uncorrected. Nuclear magnetic resonance (NMR) spectroscopic analysis was performed on a Bruker DRX-400 spectrometer at 400 MHz for ^1^H-NMR and 100 MHz for ^13^C-NMR. Chemical shifts are reported in δ ppm (parts per million) relative to TMS (0.00) as internal standard. Splitting parterns were assigned as s (singlet), d (doublet), t (triplet), q (quartet), m (multiplet), or br s (broad singlet). Mass spectra (ESI) were performed on an Agilent 1100s mass spectrometer, and the values are reported in *m/z*.

### 3.2. Synthesis of 2-(3,4-Diacetoxyphenyl)-4-oxo-4H-chromene-3,5,7-triyl triacetate (**5**)

Quercetin (**2**, 1.0 g, 3.0 mmol), acetic anhydride (6.1 g, 60.0 mmol), and pyridine (15 mL) were heated to reflux under argon atmosphere for 6 h. After cooling down to room temperature, the mixture was poured into 50 mL of ice water and filtered to give the product **5** in 79% yield. ^1^H-NMR (DMSO-*d_6_*): δ 7.85–7.88 (m, 2H, -ArH), 7.65 (d, *J* = 2.0 Hz, 1H, -ArH), 7.53 (d, *J* = 8.0 Hz, 1H, -ArH), 7.18 (d, *J* = 2.0 Hz, 1H, -ArH), 2.32–2.34 (m, 15H, -CH_3_-C=O); ESI-MS (*m/z*) 513.1 [M+H]^+^.

### 3.3. Synthesis of 4-(3,5-Diacetoxy-7-hydroxy-4-oxo-4H-chromen-2-yl)-1,2-phenylene diacetate (**6**)

To a solution of **5** (1.0 g, 2.13 mmol) in anhydrous NMP (30 mL) at 0 °C was added imidazole (50 mg, 0.74 mmol) followed by thiophenol (0.22 mL, 2.13 mmol). After stirring at 25 °C until the disappearance of the starting material as monitored by TLC, the mixture was diluted with CH_2_Cl_2_, and then washed succesively with 1 N aq. HCl and saturated brine. The organic phase was dried over anhydrous Na_2_SO_4_ and concentrated. The residue was purified by silica gel column chromatography (CH_2_Cl_2_/MeOH, 50/1, v/v) to give compound **6** as white powder in 70% yield. ^1^H-NMR (DMSO*-d_6_*): δ 7.78–7.80 (m, 2H, -ArH), 7.48 (d, *J* = 8.2 Hz, 1H, -ArH), 6.84 (d, *J* = 2.3 Hz, 1H, -ArH), 6.51 (d, *J* = 2.3 Hz, 1H, -ArH), 2.27–2.30 (m, 12H, -CH_3_-C=O); ESI-MS (*m/z*): 470.1 [M+H]^+^.

### 3.4. General Procedure for the Preparation of **7a**–**7s**

K_2_CO_3_ (95 mg, 0.69 mmol) and appropriate benzyl bromide (0.35 mmol) were added to solution of **6** (200 mg, 0.23 mmol) in acetone (15 mL). The reaction mixture was stirred for 3 h at room temperature and then filtered. The filtrate was concentrated under reduced pressure to offer the crude product **7a**–**7s** for the next step directly without further purification.

### 3.5. General Procedure for the Preparation of **3a**–**3s**

The solution of crude compound **7a**–**7s** in NH_3_/MeOH (10 mL) was stirred for 1 h at 0 °C and concentrated under reduced pressure. This residue was purified by silica gel column chromatography (CH_2_Cl_2_/MeOH, 50/1, v/v) to give the desired compound **3a**–**3s**.

*7-(Benzyloxy)-2-(3,4-dihydroxyphenyl)-3,5-dihydroxy-4H-chromen-4-one* (**3a**). Yield 50%. m.p. 255–257 °C; ^1^H-NMR (DMSO-*d_6_*) δ 7.69 (d, *J* = 2.0 Hz, 1H, -ArH), 7.54 (dd, *J* = 8.6, 2.0 Hz, 1H, -ArH), 7.35–7.46 (m, 5H, -ArH) 6.88 (d, *J* = 8.6 Hz, 1H, -ArH), 6.78 (d, *J* = 2.0 Hz, 1H, -ArH), 6.41 (d, *J* = 2.0 Hz, 1H, -ArH), 5.21 (s, 2H, -CH_2_); ^13^C-NMR (DMSO-*d_6_*) δ 176.4, 164.3, 160.9, 156.4, 148.3, 147.8, 145.5, 136.7, 136.5, 129.0 (2C), 128.6, 128.3 (2C), 122.3, 120.5, 116.0, 115.7, 104.6, 98.5, 93.2, 70.4; ESI-MS (*m/z*): 392.8 [M+H]^+^, 412.9 [M+Na]^+^.

*2-(3,4-Dihydroxyphenyl)-7-((2-fluorobenzyl)oxy)-3,5-dihydroxy-4H-chromen-4-one* (**3b**). Yield 51%. m.p. 254–255 °C; ^1^H-NMR (DMSO-*d_6_*) δ 12.52 (s, 1H, -OH), 9.68 (s, 1H, -OH), 9.56 (s, 1H, -OH), 9.34 (s, 1H, -OH), 7.74 (d, *J* = 2.0 Hz, 1H, -ArH), 7.68–7.58 (m, 1H, -ArH), 7.58 (dd, *J* = 8.4, 2.0 Hz, 1H, -ArH), 7.44–7.49 (m, 1H, -ArH), 7.25–7.32 (m, 2H, -ArH), 6.90 (d, *J* = 8.4 Hz, 1H, -ArH), 6.86 (d, *J* = 2.0 Hz, 1H, -ArH), 6.55 (d, *J* = 2.0 Hz, 1H, -ArH), 5.27 (s, 2H, -CH_2_); ^13^C-NMR (DMSO-*d_6_*) δ 176.4, 164.1, 160.9 (d, *J*_C-F_ = 245.1 Hz), 160.9, 156.4, 148.3, 147.8, 145.5, 136.6, 131.4 (d, *J*_C-F_ = 3.8 Hz), 131.2 (d, *J*_C-F_ = 8.3 Hz), 125.1 (d, *J*_C-F_ = 3.3 Hz), 123.5 (d, *J*_C-F_ = 14.5 Hz), 122.3, 120.5, 116.0 (d, *J*_C-F_ = 20.6 Hz), 116.0, 115.7, 104.7, 98.4, 93.1, 64.8 (d, *J*_C-F_ = 3.6 Hz); ESI-MS (*m/z*): 411.1 [M+H]^+^, 433.0 [M+Na]^+^.

*7-((2-Chlorobenzyl)oxy)-2-(3,4-dihydroxyphenyl)-3,5-dihydroxy-4H-chromen-4-one* (**3c**). Yield 49%. m.p. 253–254 °C; ^1^H-NMR (DMSO-*d_6_*) δ 12.52 (s, 1H, -OH), 9.67 (brs, 1H, -OH), 9.54 (brs, 1H, -OH), 9.32 (s, 1H, -OH), 7.74 (d, *J* = 2.0 Hz, 1H, -ArH), 7.63–7.65 (m, 1H, -ArH), 7.58 (dd, *J* = 8.4, 2.0 Hz, 1H, -ArH), 7.54–7.56 (m, 1H, -ArH), 7.40–7.46 (m, 2H, -ArH), 6.90 (d, *J* = 8.4 Hz, 1H, -ArH), 6.86 (d, *J* = 2.4 Hz, 1H, -ArH), 6.45 (d, *J* = 2.4 Hz, 1H, -ArH), 5.29 (s, 2H, -CH_2_); ^13^C-NMR (DMSO-*d_6_*) δ 175.8, 163.6, 160.3, 155.9, 147.8, 147.3, 145.0, 136.0, 133.4, 132.8, 130.4, 130.1, 129.4, 127.4, 121.7, 119.9, 115.5, 115.2, 104.2, 97.8, 92.6, 67.5; ESI-MS (*m/z*): 427.0 [M+H]^+^.

*7-((2-Bromobenzyl)oxy)-2-(3,4-dihydroxyphenyl)-3,5-dihydroxy-4H-chromen-4-one* (**3d**). Yield 44%. m.p. 248–249 °C; ^1^H-NMR (DMSO-*d_6_*) δ 12.53 (s, 1H, -OH), 9.67 (brs, 1H, -OH), 9.55 (brs, 1H, -OH), 9.33 (s, 1H, -OH), 7.74 (d, *J* = 2.4 Hz, 1H, -ArH), 7.72 (dd, *J* = 8.0, 1.2 Hz, 1H, -ArH), 7.63 (dd, *J* = 7.6, 1.6 Hz, 1H, -ArH), 7.58 (dd, *J* = 8.4, 2.4 Hz, 1H, -ArH), 7.46 (td, *J* = 7.6, 1.2 Hz, 1H, -ArH), 7.35 (td, *J* = 7.6, 1.6 Hz, 1H, -ArH), 6.90 (d, *J* = 8.4 Hz, 1H, -ArH), 6.86 (d, *J* = 2.0 Hz, 1H, -ArH), 6.45 (d, *J* = 2.0 Hz, 1H, -ArH), 5.25 (s, 2H, -CH_2_); ^13^C-NMR (DMSO-*d_6_*) δ 176.4, 164.1, 160.9, 156.5, 148.3, 147.9, 145.5, 136.6, 135.5, 133.2, 131.1, 130.0, 128.5, 123.7, 122.3, 120.5, 116.0, 115.7, 104.8, 98.4, 93.1, 70.3; ESI-MS (*m/z*): 471.0, 473.0 [M+H]^+^.

*2-(3,4-Dihydroxyphenyl)-3,5-dihydroxy-7-((2-methylbenzyl)oxy)-4H-chromen-4-one* (**3e**). Yield 47%. m.p. 241–242 °C; ^1^H-NMR (DMSO-*d_6_*) δ 12.52 (s, 1H, -OH), 9.66 (s, 1H, -OH), 9.53 (s, 1H, -OH), 9.33 (s, 1H, -OH) 7.74 (d, *J* = 2.4 Hz, 1H, -ArH), 7.58 (dd, *J* = 8.4, 2.4 Hz, 1H, -ArH), 7.44 (d, *J* = 8.8 Hz, 1H, -ArH), 7.21–7.30 (m, 3H, -ArH), 6.90 (d, *J* = 8.4 Hz, 1H, -ArH), 6.85 (d, *J* = 2.0 Hz, 1H, -ArH), 6.46 (d, *J* = 2.0 Hz, 1H, -ArH), 5.22 (s, 2H, -CH_2_), 2.34 (s, 3H, -CH_3_); ^13^C-NMR (DMSO-*d_6_*) δ 175.9, 164.0, 160.4, 156.0, 147.8, 147.3, 145.1, 136.8, 134.1, 130.2, 128.6, 128.3, 125.8, 121.8, 120.0, 115.5, 115.2, 104.1, 97.9, 92.7, 68.7, 18.4; ESI-MS (*m/z*): 407.0 [M+H]^+^, 429.1 [M+Na]^+^.

*2-(((2-(3,4-Dihydroxyphenyl)-3,5-dihydroxy-4-oxo-4H-chromen-7-yl)oxy)methyl)benzonitrile* (**3f**). Yield 40%. m.p. 248–249 °C; ^1^H-NMR (DMSO-*d_6_*) δ 12.54 (s, 1H, -OH), 9.68 (s, 1H, -OH), 9.56 (s, 1H, -OH), 9.33 (s, 1H, -OH), 7.95 (d, *J* = 7.6 Hz, 1H, -ArH), 7.78–7.81 (m, 2H, -ArH), 7.74 (d, *J* = 2.0 Hz, 1H, -ArH), 7.57–7.63 (m, 2H, -ArH), 6.90 (d, *J* = 7.6 Hz, 1H, -ArH), 6.89 (d, *J* = 2.0 Hz, 1H, -ArH), 6.47 (d, *J* = 2.0 Hz, 1H, -ArH), 5.39 (s, 2H, -CH_2_); ^13^C-NMR (DMSO-*d_6_*) δ 176.4, 163.9, 160.9, 156.4, 148.4, 147.9, 145.6, 139.6, 136.6, 134.0, 133.9, 129.9, 122.3, 120.5, 117.6, 116.0, 115.7, 111.9, 104.9, 98.4, 93.3, 68.7; ESI-MS (*m/z*): 417.7 [M+H]^+^, 856.5 [2M+Na]^+^.

*2-(3,4-Dihydroxyphenyl)-3,5-dihydroxy-7-((2-nitrobenzyl)oxy)-4H-chromen-4-one* (**3g**). Yield 43%. m.p. 260–261 °C; ^1^H-NMR (DMSO-*d_6_*) δ 12.53 (s, 1H, -OH), 9.66 (s, 1H, -OH), 9.54 (brs, 1H, -OH), 9.32 (s, 1H, -OH), 8.17 (d, *J* = 8.4 Hz, 1H, -ArH), 7.78–7.82 (m, 2H, -ArH), 7.73 (d, *J* = 2.0 Hz, 1H, -ArH), 7.63–7.67 (m, 1H, -ArH), 7.58 (dd, *J* = 8.8, 2.0 Hz, 1H, -ArH), 6.89 (d, *J* = 8.8 Hz, 1H, -ArH), 6.83 (d, *J* = 2.4 Hz, 1H, -ArH), 6.50 (d, *J* = 2.4 Hz, 1H, -ArH), 5.61 (s, 2H, -CH_2_); ^13^C-NMR (DMSO-*d_6_*) δ 176.4, 163.8, 160.9, 154.6, 148.3, 147.9, 145.5, 136.6, 134.6, 132.2, 129.8, 129.6, 125.4, 122.3, 120.5, 116.0, 115.7, 104.9, 98.4, 93.2, 67.5; ESI-MS (*m/z*): 438.1 [M+H]^+^.

*2-(3,4-Dihydroxyphenyl)-7-((3-fluorobenzyl)oxy)-3,5-dihydroxy-4H-chromen-4-one* (**3h**). Yield 54%. m.p. 243–245 °C; ^1^H-NMR (DMSO-*d_6_*) δ 12.52 (s, 1H, -OH), 9.67 (s, 1H, -OH), 9.54 (s, 1H, -OH), 9.33 (s, 1H, -OH), 7.72 (d, *J* = 2.4 Hz, 1H, -ArH), 7.56 (dd, *J* = 8.4, 2.4 Hz, 1H, -ArH), 7.45–7.48 (m, 1H, -ArH), 7.31–7.33 (m, 2H, -ArH), 7.20–7.22 (m, 1H, -ArH), 6.90 (d, *J* = 8.4 Hz, 1H, -ArH), 6.81 (d, *J* = 2.0 Hz, 1H, -ArH), 6.45 (d, *J* = 2.0 Hz, 1H, -ArH), 5.27 (s, 2H, -CH_2_); ^13^C-NMR (DMSO-*d_6_*) δ 176.4, 164.1, 162.7 (d, *J*_C-F_ = 242.2Hz), 160.9, 156.4, 148.3, 147.9, 145.6, 139.6 (d, *J*_C-F_ = 7.5 Hz), 136.5, 131.8 (d, *J*_C-F_ = 8.3 Hz), 124.1 (d, *J*_C-F_ = 2.8 Hz), 122.3, 120.5, 116.0, 115.7, 115.3 (d, *J*_C-F_ = 20.6 Hz), 114.8 (d, *J*_C-F_ = 21.8 Hz), 104.7, 98.5, 93.3, 69.5; ESI-MS (*m/z*): 411.1 [M+H]^+^.

*7-((3-Chlorobenzyl)oxy)-2-(3,4-dihydroxyphenyl)-3,5-dihydroxy-4H-chromen-4-one* (**3i**). Yield 40%. m.p. 260–261 °C; ^1^H-NMR (DMSO-*d_6_*) δ 12.52 (s, 1H, -OH), 9.67 (brs, 1H, -OH), 9.54 (brs, 1H, -OH), 9.32 (s, 1H, -OH), 7.73 (d, *J* = 2.4 Hz, 1H, -ArH), 7.55–7.58 (m, 2H, -ArH), 7.41–7.45 (m, 3H, -ArH), 6.90 (d, *J* = 8.4 Hz, 1H, -ArH), 6.81 (d, *J* = 2.0 Hz, 1H, -ArH), 6.46 (d, *J* = 2.0 Hz, 1H, -ArH), 5.26 (s, 2H, -CH_2_); ESI-MS (*m/z*): 427.0 [M+H]^+^.

*7-((3-Bromobenzyl)oxy)-2-(3,4-dihydroxyphenyl)-3,5-dihydroxy-4H-chromen-4-one* (**3j**). Yield 47%. m.p. 234–236 °C; ^1^H-NMR (DMSO-*d_6_*) δ 12.52 (s, 1H, -OH), 9.68 (brs, 1H, -OH), 9.55 (brs, 1H, -OH), 9.33 (s, 1H, -OH), 7.72 (d, *J* = 2.4 Hz, 1H, -ArH), 7.69 (s, 1H, -ArH), 7.56 (dd, *J* = 8.4, 2.0 Hz, 2H, -ArH), 7.49 (d, *J* = 8.0 Hz, 1H, -ArH), 7.39 (t, *J* = 8.0 Hz, 1H, -ArH), 6.90 (d, *J* = 8.8 Hz, 1H, -ArH), 6.81 (d, *J* = 2.0 Hz, 1H, -ArH), 6.46 (d, *J* = 2.0 Hz, 1H, -ArH), 5.25 (s, 2H, -CH_2_); ^13^C-NMR (DMSO-*d_6_*) δ 176.4, 164.1, 160.9, 156.4, 148.3, 147.8, 145.5, 139.5, 136.5, 131.4, 131.3, 130.8, 127.2, 122.3, 122.2, 120.5, 116.0, 115.7, 104.7, 98.5, 93.3, 69.4; ESI-MS (*m/z*): 471.0, 473.0 [M+H]^+^.

*2-(3,4-Dihydroxyphenyl)-3,5-dihydroxy-7-((3-methylbenzyl)oxy)-4H-chromen-4-one* (**3k**). Yield 43%. m.p. 224–226 °C; ^1^H-NMR (DMSO-*d_6_*) δ 12.51 (s, 1H, -OH), 9.63 (brs, 1H, -OH), 9.53 (brs, 1H, -OH), 9.33 (brs, 1H, -OH), 7.73 (d, *J* = 2.0 Hz, 1H, -ArH), 7.57 (d, *J* = 8.8, 2.0 Hz, 1H, -ArH), 7.16–7.32 (m, 4H, -ArH), 6.90 (d, *J* = 8.8 Hz, 1H, -ArH), 6.79 (d, *J* = 2.0 Hz, 1H, -ArH), 6.43 (d, *J* = 2.0 Hz, 1H, -ArH), 5.19 (s, 2H, -CH_2_), 2.33 (s, 3H, -CH_3_); ^13^C-NMR (DMSO-*d_6_*) δ 176.4, 164.4, 160.9, 156.4, 148.3, 147.8, 145.5, 138.2, 136.6, 136.5, 129.2, 128.9, 128.9, 125.4, 122.3, 120.5, 116.0, 115.7, 104.6, 98.5, 93.2, 70.5, 21.5; ESI-MS (*m/z*): 407.0 [M+H]^+^.

*3-(((2-(3,4-Dihydroxyphenyl)-3,5-dihydroxy-4-oxo-4H-chromen-7-yl)oxy)methyl)benzonitrile* (**3l**). Yield 42%. m.p. 246–247 °C; ^1^H-NMR (DMSO-*d_6_*) δ 12.52 (s, 1H, -OH), 9.66 (s, 1H, -OH), 9.53 (s, 1H, -OH), 9.31 (s, 1H, -OH), 7.96 (brs, 1H, -ArH), 7.82–7.86 (m, 2H, -ArH), 7.72 (d, *J* = 2.0 Hz, 1H, -ArH), 7.63–7.67 (m, 1H, -ArH), 7.56 (dd, *J* = 8.4, 2.0 Hz, 1H, -ArH), 6.90 (d, *J* = 8.4 Hz, 1H, -ArH), 6.83 (d, *J* = 1.6 Hz, 1H, -ArH), 6.47 (d, *J* = 1.6 Hz, 1H, -ArH), 5.31 (s, 2H, -CH_2_); ESI-MS (*m/z*): 418.1 [M+H]^+^, 440.0 [M+Na]^+^.

*2-(3,4-Dihydroxyphenyl)-3,5-dihydroxy-7-((3-nitrobenzyl)oxy)-4H-chromen-4-one* (**3m**). Yield 46%. m.p. 264–266 °C; ^1^H-NMR (DMSO-*d_6_*) δ 12.53 (s, 1H, -OH), 9.67 (s, 1H, -OH), 9.54 (brs, 1H, -OH), 9.31 (s, 1H, -OH), 8.35 (s, 1H, -ArH), 8.23 (dd, *J* = 8.4, 1.6 Hz, 1H, -ArH), 7.94 (d, *J* = 8.0 Hz, 1H, -ArH), 7.72–7.76 (m, 2H, -ArH), 7.56 (dd, *J* = 8.4, 2.0 Hz, 1H, -ArH), 6.90 (d, *J* = 8.4 Hz, 1H, -ArH), 6.85 (d, *J* = 2.4 Hz, 1H, -ArH), 6.49 (d, *J* = 2.4 Hz, 1H, -ArH), 5.41 (s, 2H, -CH_2_); ^13^C-NMR (DMSO-*d_6_*) δ 175.9, 163.4, 160.5, 155.9, 147.8, 147.4, 145.1, 138.6, 136.1, 134.2, 130.2, 123.0, 122.2, 121.8, 120.0, 115.5, 115.2, 104.3, 98.0, 92.9, 68.5; ESI-MS (*m/z*): 438.1 [M+H]^+^.

*2-(3,4-Dihydroxyphenyl)-7-((4-fluorobenzyl)oxy)-3,5-dihydroxy-4H-chromen-4-one* (**3n**). Yield 50%. m.p.254–255 °C; ^1^H-NMR (DMSO-*d_6_*) δ 12.51 (s, 1H, -OH), 9.68 (s, 1H, -OH), 9.53 (brs, 1H, -OH), 9.33 (s, 1H, -OH) 7.72 (d, *J* = 2.0 Hz, 1H, -ArH), 7.52–7.58 (m, 3H, -ArH), 7.27 (d, *J* = 8.8 Hz, 1H, -ArH), 7.24 (d, *J* = 8.8 Hz, 1H, -ArH), 6.90 (d, *J* = 8.4 Hz, 1H, -ArH), 6.81 (d, *J* = 2.4 Hz, 1H, -ArH), 6.43 (d, *J* = 2.4 Hz, 1H, -ArH), 5.22 (s, 2H, -CH_2_); ^13^C-NMR (DMSO-*d_6_*) δ 175.9, 163.7, 161.8 (d, *J*_C-F_ = 244.4 Hz), 160.0, 155.9, 147.8, 147.3, 145.0, 136.0, 132.4 (d, *J*_C-F_ = 2.9 Hz), 130.2 (d, *J*_C-F_ = 8.3 Hz, 2C), 121.8, 120.0, 115.5, 115.4(d, *J* = 21.4 Hz, 2C), 115.2, 104.1, 98.0, 92.7, 69.2; ESI-MS (*m/z*): 411.1 [M+H]^+^.

*7-((4-Chlorobenzyl)oxy)-2-(3,4-dihydroxyphenyl)-3,5-dihydroxy-4H-chromen-4-one* (**3o**). Yield 41%. m.p. 266–267 °C; ^1^H-NMR (DMSO-*d_6_*) δ 12.51 (s, 1H, -OH), 9.67 (s, 1H, -OH), 9.53 (s, 1H, -OH), 9.32 (s, 1H, -OH), 7.71 (d, *J* = 2.0 Hz, 1H, -ArH), 7.56 (dd, *J* = 8.4, 2.0 Hz, 1H, -ArH), 7.47–7.52 (m, 4H, -ArH), 6.90 (d, *J* = 8.4 Hz, 1H, -ArH), 6.80 (d, *J* = 2.0 Hz, 1H, -ArH), 6.44 (d, *J* = 2.0 Hz, 1H, -ArH), 5.24 (s, 2H, -CH_2_); ESI-MS (*m/z*): 427.0 [M+H]^+^.

*7-((4-Bromobenzyl)oxy)-2-(3,4-dihydroxyphenyl)-3,5-dihydroxy-4H-chromen-4-one* (**3p**). Yield 44%. m.p.256–257 °C; 1H-NMR (DMSO-*d_6_*) δ 12.51 (s, 1H, -OH), 9.68 (brs, 1H, -OH), 9.54 (brs, 1H, -OH), 9.34 (brs, 1H, -OH), 7.72 (d, *J* = 2.0 Hz, 1H, -ArH), 7.62 (d, *J* = 8.4 Hz, 2H, -ArH), 7.56 (dd, *J* = 8.8, 2.0 Hz, 1H, -ArH), 7.44 (d, *J* = 8.4 Hz, 2H, -ArH), 6.90 (d, *J* = 8.8 Hz, 1H, -ArH), 6.80 (d, *J* = 2.4 Hz, 1H, -ArH), 6.43 (d, *J* = 2.4 Hz, 1H, -ArH), 5.23 (s, 2H, -CH_2_); ^13^C-NMR (DMSO-*d_6_*) δ 176.4, 164.1, 160.9, 156.4, 148.3, 147.8, 145.5, 136.5, 136.2, 131.9 (2C), 130.4 (2C), 122.3, 121.7, 120.5, 116.0, 115.7, 104.7, 98.5, 93.3, 69.5; ESI-MS (m/z): 471.0, 473.0 [M+H]^+^.

*2-(3,4-Dihydroxyphenyl)-3,5-dihydroxy-7-((4-methylbenzyl)oxy)-4H-chromen-4-one* (**3q**). Yield 56%. m.p. 261–264 °C; ^1^H-NMR (DMSO-*d_6_*) δ 12.51 (s, 1H, -OH), 9.51–9.43 (brs, 3H, -OH), 7.73 (brs, 1H, -ArH), 7.57 (d, *J* = 8.4 Hz, 1H, -ArH), 7.36 (d, *J* = 8.0 Hz, 2H, -ArH), 7.22 (d, *J* = 8.0 Hz, 2H, -ArH), 6.90 (d, *J* = 8.4 Hz, 1H, -ArH), 6.78 (d, *J* = 1.6 Hz, 1H, -ArH), 6.41 (d, *J* = 1.6 Hz, 1H, -ArH), 5.18 (s, 2H, -CH_2_), 2.31 (s, 3H, -CH_3_); ^13^C-NMR (DMSO-*d_6_*) δ 175.9, 163.9, 160.4, 155.9, 147.9, 147.3, 145.1, 137.4, 136.0, 133.1, 129.0 (2C), 127.9 (2C), 121.8, 120.0, 115.5, 115.2, 104.1, 98.0, 92.7, 69.8, 20.8; ESI-MS (*m/z*): 407.0 [M+H]^+^.

*4-(((2-(3,4-Dihydroxyphenyl)-3,5-dihydroxy-4-oxo-4H-chromen-7-yl)oxy)methyl)benzonitrile* (**3r**). Yield 45%. m.p. 252–254 °C; ^1^H-NMR (DMSO-*d_6_*) δ 12.52 (s, 1H, -OH), 9.68 (s, 1H, -OH), 9.55 (s, 1H, -OH), 9.32 (s, 1H, -OH), 7.90 (d, *J* = 8.0 Hz, 2H, -ArH), 7.72 (d, *J* = 1.6 Hz, 1H, -ArH), 7.67 (d, *J* = 8.0 Hz, 2H, -ArH), 7.56 (dd, *J* = 8.4, 1.6 Hz, 1H, -ArH), 6.90 (d, *J* = 8.4 Hz, 1H, -ArH), 6.81 (d, *J* = 1.6 Hz, 1H, -ArH), 6.46 (d, *J* = 1.6 Hz, 1H, -ArH), 5.37 (s, 2H, -CH_2_); ^13^C-NMR (DMSO-*d_6_*) δ 176.4, 163.9, 160.9, 156.4, 148.4, 147.9, 145.6, 142.5, 136.6, 133.0(2C), 128.6(2C), 122.3, 120.5, 119.2, 116.0, 115.7, 111.2, 104.8, 98.5, 93.3, 69.3; ESI-MS (*m/z*): 417.9 [M+H]^+^.

*2-(3,4-Dihydroxyphenyl)-3,5-dihydroxy-7-((4-nitrobenzyl)oxy)-4H-chromen-4-one* (**3s**). Yield 47%. m.p. 253.5–254.5 °C; ^1^H-NMR (DMSO-*d_6_*) δ 12.53 (s, 1H, -OH), 9.68 (s, 1H, -OH), 9.55 (brs, 1H, -OH), 9.32 (s, 1H, -OH), 8.29 (d, *J* = 8.8 Hz, 2H, -ArH), 7.75 (d, *J* = 8.8 Hz, 2H, -ArH), 7.72 (d, *J* = 2.0 Hz, 1H, -ArH), 7.56 (dd, *J* = 8.4, 2.0 Hz, 1H, -ArH), 6.90 (d, *J* = 8.4 Hz, 1H, -ArH), 6.83 (d, *J* = 2.4 Hz, 1H, -ArH), 6.47 (d, *J* = 2.4 Hz, 1H, -ArH), 5.43 (s, 2H, -CH_2_); ^13^C-NMR (DMSO-*d_6_*) δ 176.4, 163.9, 161.0, 156.4, 148.4, 147.9, 147.6, 145.6, 144.6, 136.6, 128.8 (2C), 124.2 (2C), 122.3, 120.5, 116.0, 115.7, 104.8, 98.5, 93.4, 69.1, 23.0; ESI-MS (*m/z*): 438.1 [M+H]^+^.

### 3.6. Synthesis of 7-(Benzyloxy)-2-(3,4-bis(benzyloxy)phenyl)-3,5-dihydroxy-4H-chromen-4-one (**9**)

A mixture of rutin (**8**, 5.00 g, 8.2 mmol) and potassium carbonate (2.83 g, 20.5 mmol) in dry DMF (40 mL) was stirred under argon atmosphere for 0.5 h. Benzyl bromide (3.2 mL, 27.1 mmol) was then added dropwise. After stirring for 3 h at 60 °C, the mixture was acidified to pH 5 with 10% aqueous acetic acid solution, and the precipitate was collected by centrifugation. Ethanol (60 mL) was added to the precipitate and then conc. aqueous hydrochloric acid (9 mL) of was add in portions. This reaction mixture was stirred at 70 °C for 2 h. After cooling to room temperature, the precipitate was filtered and washed with water. The crude product was recrystallized from CH_2_Cl_2_/EtOH to afford **9** as yellow powder in 85% yield, m.p. 186–188 °C. ^1^H-NMR (DMSO-*d_6_*) δ 7.90 (d, *J* = 2.0 Hz, 1H, -ArH), 7.84 (dd, *J* = 8.8, 2.0 Hz, 1H, -ArH), 7.53–7.30 (m, 15H, -ArH), 7.25 (d, *J* = 8.8 Hz, 1H, -ArH), 6.86 (d, *J* = 2.0 Hz, 1H, -ArH) 6.45 (d, *J* = 2.0 Hz, 1H, -ArH), 5.24 (s, 4H, -CH_2_), 5.21 (s, 2H, -CH_2_); ESI-MS (*m/z*) 573.1 [M+H]^+^.

### 3.7. General Procedure for the Preparation of **10a**–**10o**

A mixture of compound **9** (200 mg, 0.35 mmol), EDCI (100 mg, 0.525 mmol), DMAP (9 mg, 0.07 mmol) and appropriate benzoic acid (0.35 mmol) in DMF (20 mL) was stirred at 30 °C overnight. The mixture was poured into 100 mL of water and filtered to give the crude products **10a**–**10o** for the next step directly without further purification.

### 3.8. General Procedure for the Preparation of **4a**–**4o**

To a solution of the appropriate benzyl-protected quercetin-3-*O*-ester derivatives **10a**–**10o** in ethanol and 1,4-dioxane (20 mL, 3:1) was added 10% palladium on carbon (20 mg). The mixture was stirred under hydrogen atmosphere at room temperature for 6 h. The resulting mixture was filtered, washed with EtOH and purified by silica gel column chromatography (CH_2_Cl_2_/MeOH, 20/1, v/v) to give the desired compounds **4a**–**4o**.

*2-(3,4-Dihydroxyphenyl)-5,7-dihydroxy-4-oxo-4H-chromen-3-yl benzoate* (**4a**). Yield 54%. m.p. 173–174 °C; ^1^H-NMR (DMSO-*d_6_*): δ 12.16 (s, 1H, -OH), 11.05 (brs, 1H, -OH), 9.90 (brs, 1H, -OH), 9.47 (brs, 1H, -OH), 8.15 (d, *J* = 7.9 Hz, 2H, -ArH), 7.80 (t, *J* = 7.9 Hz, 1H, -ArH), 7.64 (dd, *J* = 7.9, 7.9 Hz, 2H, -ArH), 7.36 (d, *J* = 2.4 Hz, 1H, -ArH), 7.31 (dd, *J* = 8.4, 2.4 Hz, 1H, -ArH), 6.86 (d, *J* = 8.4 Hz, 1H, -ArH), 6.52 (d, *J* = 2.0 Hz, 1H, -ArH), 6.28 (d, *J* = 2.0 Hz, 1H, -ArH); ESI-MS (*m/z*) 407.0 [M+H]^+^.

*2-(3,4-Dihydroxyphenyl)-5,7-dihydroxy-4-oxo-4H-chromen-3-yl 2-fluorobenzoate* (**4b**). Yield 54%. m.p. 186–187 °C; ^1^H-NMR (DMSO-*d_6_*): δ 12.13 (s, 1H, -OH), 11.14 (brs, 1H, -OH), 10.00 (brs, 1H, -OH), 9.54 (brs, 1H, -OH), 8.14 (ddd, *J* = 7.6, 7.6, 2.0 Hz, 1H, -ArH), 7.86–7.82 (m, 1H, -ArH), 7.51–7.46 (m, 2H, -ArH), 7.38 (d, *J* = 2.4 Hz, 1H, -ArH), 7.34 (dd, *J* = 8.4, 2.4 Hz, 1H, -ArH), 6.91 (d, *J* = 8.4 Hz, 1H, -ArH), 6.52 (d, *J* = 2.0 Hz, 1H, -ArH), 6.28 (d, *J* = 2.0 Hz, 1H, -ArH); ESI-MS (*m/z*) 425.0 [M+H]^+^.

*2-(3,4-Dihydroxyphenyl)-5,7-dihydroxy-4-oxo-4H-chromen-3-yl 2-aminobenzoate* (**4c**). Yield 58%. m.p. 198–199 °C; ^1^H-NMR (DMSO-*d_6_*): δ 12.24 (s, 1H, -OH), 11.02 (brs, 1H, -OH), 9.91 (brs, 1H, -OH), 9.45 (brs, 1H, -OH), 7.95 (dd, *J* = 8.0, 2.0 Hz, 1H, -ArH), 7.38–7.34 (m, 2H, -ArH), 7.30 (dd, *J* = 8.4, 2.4 Hz, 1H, -ArH), 6.85 (d, *J* = 8.4 Hz, 1H, -ArH), 6.84 (d, *J* = 7.6 Hz, 1H, -ArH), 6.74 (brs, 2H, -ArH), 6.62 (dd, *J* = 7.6, 7.6 Hz, 1H, -ArH), 6.51 (d, *J* = 2.0 Hz, 1H, -ArH), 6.26 (d, *J* = 2.0 Hz, 1H, -ArH); ESI-MS (*m/z*) 422.0 [M+H]^+^.

*2-(3,4-Dihydroxyphenyl)-5,7-dihydroxy-4-oxo-4H-chromen-3-yl 2-methoxybenzoate* (**4d**). Yield 52%. m.p. 183–184 °C; ^1^H-NMR (DMSO-*d_6_*): δ 12.23 (s, 1H, -OH), 11.11 (brs, 1H, -OH), 10.00 (brs, 1H, -OH), 9.48 (brs, 1H, -OH), 7.98 (dd, *J* = 7.6, 2.0 Hz, 1H, -ArH), 7.69 (ddd, *J* = 8.8, 7.6, 2.0 Hz, 1H, -ArH), 7.41 (d, *J* = 2.4 Hz, 1H, -ArH), 7.38 (dd, *J* = 8.4, 2.4 Hz, 1H, -ArH), 7.26 (d, *J* = 8.8 Hz, 1H, -ArH), 7.13 (dd, *J* = 7.6, 7.6 Hz, 1H, -ArH), 6.90 (d, *J* = 8.4 Hz, 1H, -ArH), 6.53 (d, *J* = 2.0 Hz, 1H, -ArH), 6.29 (d, *J* = 2.0 Hz, 1H, -ArH), 3.88 (s, 3H, -CH_3_); ESI-MS (*m/z*) 437.0 [M+H]^+^.

*2-(3,4-Dihydroxyphenyl)-5,7-dihydroxy-4-oxo-4H-chromen-3-yl 2-cyanobenzoate* (**4e**). Yield 57%. m.p. 196–197 °C; ^1^H-NMR (DMSO-*d_6_*): δ 12.24 (s, 1H, -OH), 11.02 (brs, 1H, -OH), 9.91 (brs, 1H, -OH), 9.45 (brs, 1H, -OH), 8.45–8.43 (m, 1H, -ArH), 8.17–8.14 (m, 1H, -ArH), 8.00–7.97 (m, 2H, -ArH), 7.38 (d, *J* = 2.4 Hz, 1H, -ArH), 7.35 (dd, *J* = 8.4, 2.4 Hz, 1H, -ArH), 6.89 (d, *J* = 8.4 Hz, 1H, -ArH), 6.56 (d, *J* = 2.0 Hz, 1H, -ArH), 6.31 (d, *J* = 2.0 Hz, 1H, -ArH); ESI-MS (*m/z*) 432.0 [M+H]^+^, 454.0 [M+Na]^+^.

*2-(3,4-Dihydroxyphenyl)-5,7-dihydroxy-4-oxo-4H-chromen-3-yl 3-fluorobenzoate* (**4f**). Yield 52%. m.p. 211–213 °C; ^1^H-NMR (DMSO-*d_6_*) δ 12.11 (s, 1H, -OH), 11.07 (brs, 1H, -OH), 9.92 (brs, 1H, -OH), 9.50 (brs, 1H, -OH), 8.01 (ddd, *J* = 6.8, 1.6, 2.0 Hz, 1H, -ArH), 7.92 (dd, *J* = 9.2, 2.0 Hz, 1H, -ArH), 7.74–7.65 (m, 2H, -ArH), 7.36 (d, *J* = 2.0 Hz, 1H, -ArH), 7.32 (dd, *J* = 8.4, 2.0 Hz, 1H, -ArH), 6.87 (d, *J* = 8.4 Hz, 1H, -ArH), 6.53 (d, *J* = 2.0 Hz, 1H, -ArH), 6.28 (d, *J* = 2.0 Hz, 1H, -ArH); ^13^C-NMR (DMSO-*d_6_*) δ 174.51, 164.73, 162.30 (d, *J*_C-F_ = 3.0 Hz), 162.05 (d, *J*_C-F_ = 244.3 Hz), 161.03, 156.66, 156.39, 149.37, 145.47, 131.50 (d, *J*_C-F_ = 7.9 Hz), 129.98 (d, *J*_C-F_ = 7.5 Hz), 129.69, 126.32 (d, *J*_C-F_ = 2.5 Hz), 121.66 (d, *J*_C-F_ = 21.1 Hz), 120.56, 119.41, 116.55 (d, *J*_C-F_ = 23.1 Hz), 115.91, 115.09, 103.40, 99.18, 94.20; ESI-MS (*m/z*) 425.0 [M+H]^+^, 871.2 [2M+Na]^+^.

*2-(3,4-Dihydroxyphenyl)-5,7-dihydroxy-4-oxo-4H-chromen-3-yl 3-aminobenzoate* (**4g**). Yield 50%. m.p. 216–217 °C; ^1^H-NMR (DMSO-*d_6_*) δ 12.20 (s, 1H, -OH), 11.03 (brs, 1H, -OH), 9.92 (brs, 1H, -OH), 9.45 (brs, 1H, -OH), 7.34 (d, *J* = 2.4 Hz, 1H, -ArH), 7.32 (d, *J* = 2.0 Hz, 1H, -ArH), 7.28 (dd, *J* = 8.4, 2.4 Hz, 1H, -ArH), 7.25–7.21 (m, 2H, -ArH), 6.91–6.89 (m, 1H, -ArH), 6.86 (d, *J* = 8.4 Hz, 1H, -ArH), 6.51 (d, *J* = 2.0 Hz, 1H, -ArH), 6.27 (d, *J* = 2.0 Hz, 1H, -ArH); ESI-MS (m/z) 422.0 [M+H]^+^.

*2-(3,4-Dihydroxyphenyl)-5,7-dihydroxy-4-oxo-4H-chromen-3-yl 3-methoxybenzoate* (**4h**). Yield 60%. m.p. 173–174 °C; ^1^H-NMR (DMSO-*d_6_*) δ 12.16 (s, 1H, -OH), 11.06 (brs, 1H, -OH), 9.92 (brs, 1H, -OH), 9.47 (brs, 1H, -OH), 7.74 (d, *J* = 7.6 Hz, 1H, -ArH), 7.59 (dd, *J* = 3.0, 2.0Hz, 1H, -ArH), 7.55 (dd, *J* = 7.6, 7.6 Hz, 1H, -ArH), 7.37–7.35 (m, 2H, -ArH), 7.30 (dd, *J* = 8.4, 2.4 Hz, 1H, -ArH), 6.86 (d, *J* = 8.4 Hz, 1H, -ArH), 6.52 (d, *J* = 2.0 Hz, 1H, -ArH), 6.27 (d, *J* = 2.0 Hz, 1H, -ArH); ESI-MS (*m/z*) 437.0 [M+H]^+^, 459.1 [M+Na]^+^, 895.2 [2M+Na]^+^.

*2-(3,4-Dihydroxyphenyl)-5,7-dihydroxy-4-oxo-4H-chromen-3-yl 3-cyanobenzoate* (**4i**). Yield 49%. m.p. 142–143 °C; ^1^H-NMR (DMSO-*d_6_*) δ 12.08 (s, 1H, -OH), 11.08 (brs, 1H, -OH), 9.92 (brs, 1H, -OH), 9.50 (s, 1H, -OH), 8.58 (dd, *J* = 2.0, 2.0 Hz, 1H, -ArH), 8.45 (ddd, *J* = 8.0, 2.0, 2.0 Hz, 1H, -ArH), 8.27 (ddd, *J* = 8.0, 2.0, 2.0 Hz, 1H, -ArH), 7.86 (d, *J* = 8.0 Hz, 1H, -ArH), 7.37 (d, *J* = 2.4 Hz, 1H, -ArH), 6.83 (dd, *J* = 8.4, 2.4 Hz, 1H, -ArH), 6.87 (d, *J* = 8.4 Hz, 1H, -ArH), 6.53 (d, *J* = 2.0 Hz, 1H, -ArH), 6.28 (d, *J* = 2.0 Hz, 1H, -ArH); ESI-MS (*m/z*) 432.1 [M+H]^+^, 454.0 [M+Na]^+^.

*2-(3,4-Dihydroxyphenyl)-5,7-dihydroxy-4-oxo-4H-chromen-3-yl 3-chlorobenzoate* (**4j**). Purified by prepared HPLC. Yield 35%. m.p. > 300 °C; ^1^H-NMR (DMSO-*d_6_*) δ 8.13 (brs, 1H, -ArH), 8.09 (d, *J* = 8.0 Hz, 1H, -ArH), 7.71 (d, *J* = 8.0 Hz, 1H, -ArH), 7.56 (dd, *J* = 8.0, 8.0 Hz, 1H, -ArH), 7.33 (brs, 1H, -ArH), 7.29 (d, *J* = 8.0 Hz, 1H, -ArH), 6.81 (d, *J* = 8.0 Hz, 1H, -ArH), 6.42 (brs, 1H, -ArH), 6.21 (brs, 1H, -ArH); ESI-MS (*m/z*) 440.8 [M+H]^+^.

*2-(3,4-Dihydroxyphenyl)-5,7-dihydroxy-4-oxo-4H-chromen-3-yl 4-fluorobenzoate* (**4k**). Yield 56%. m.p. 203–205 °C; ^1^H-NMR (DMSO-*d_6_*) δ 12.14 (s, 1H, -OH), 11.06 (brs, 1H, -OH), 9.91 (brs, 1H, -OH), 9.48 (brs, 1H, -OH), 8.24 (d, *J* = 8.8 Hz, 1H, -ArH), 8.23(d, *J* = 8.8 Hz, 1H, -ArH) 7.48 (d, *J* = 8.8 Hz, 1H, -ArH), 7.46 (d, *J* = 8.8 Hz, 1H, -ArH), 7.36 (d, *J* = 2.4 Hz, 1H, -ArH), 7.31 (dd, *J* = 8.4, 2.4 Hz, 1H, -ArH), 6.87 (d, *J* = 8.4 Hz, 1H, -ArH), 6.52 (d, *J* = 2.0 Hz, 1H, -ArH), 6.28 (d, *J* = 2.0 Hz, 1H, -ArH); ^13^C-NMR (DMSO-*d_6_*) 174.59, 165.72 (d, *J*_C-F_ = 251.8 Hz) 164.64, 162.34, 160.98, 156.59, 156.28, 149.26, 145.40, 133.06 (d, *J*_C-F_ = 9.8 Hz, 2C), 129.67, 124.36 (d, *J*_C-F_ = 2.6 Hz), 120.45, 119.43, 116.33 (d, *J*_C-F_ = 22.1 Hz, 2C), 115.81, 115.02, 103.35, 99.08, 94.10; ESI-MS (*m/z*) 425.0 [M+H]^+^, 871.2 [2M+Na]^+^.

*2-(3,4-Dihydroxyphenyl)-5,7-dihydroxy-4-oxo-4H-chromen-3-yl 4-aminobenzoate* (**4l**). The product was prepared according to procedure described above starting from **9** and 4-nitrobenzoic acid. Yield 55%. m.p. 233–234 °C; ^1^H-NMR (DMSO-*d_6_*) δ 12.30 (s, 1H, -OH), 11.00 (brs, 1H, -OH), 9.88 (brs, 1H, -OH), 9.42 (s, 1H, -OH), 7.78 (d, *J* = 8.8 Hz, 2H, -ArH), 7.35 (d, *J* = 2.0 Hz, 1H, -ArH), 7.28 (dd, *J* = 8.4, 2.0 Hz, 1H, -ArH), 6.84 (d, *J* = 8.4 Hz, 1H, -ArH), 6.63 (d, *J* = 8.8 Hz, 2H, -ArH), 6.49 (d, *J* = 2.4 Hz, 1H, -ArH), 6.26 (brs, 2H, -ArH), 6.25 (d, *J* = 2.4 Hz, 1H, -ArH); ESI-MS (*m/z*) 422.0 [M+H]^+^.

*2-(3,4-Dihydroxyphenyl)-5,7-dihydroxy-4-oxo-4H-chromen-3-yl 4-methoxybenzoate* (**4m**). Yield 50%. m.p. 194–195 °C; ^1^H-NMR (DMSO-*d_6_*) δ 12.20 (s, 1H, -OH), 11.03 (brs, 1H, -OH), 9.90 (brs, 1H, -OH), 9.44 (s, 1H, -OH), 8.10 (d, *J* = 8.8 Hz, 2H, -ArH), 7.35 (d, *J* = 2.0 Hz, 1H, -ArH), 7.29 (dd, *J* = 8.4, 2.0 Hz, 1H, -ArH), 7.14 (d, *J* = 8.8 Hz, 2H, -ArH), 6.84 (d, *J* = 8.4 Hz, 1H, -ArH), 6.51 (d, *J* = 2.0 Hz, 1H, -ArH), 6.27 (d, *J* = 2.0 Hz, 1H, -ArH), 3.89(s, 3H, -CH_3_); ESI-MS (*m/z*) 437.0 [M+H]^+^, 895.2 [2M+Na]^+^.

*2-(3,4-Dihydroxyphenyl)-5,7-dihydroxy-4-oxo-4H-chromen-3-yl 4-cyanobenzoate* (**4n**). Yield 58%. m.p. 196–197 °C; ^1^H-NMR (DMSO-*d_6_*) δ 12.07 (s, 1H, -OH), 11.08 (brs, 1H, -OH), 9.92 (brs, 1H, -OH), 9.50 (brs, 1H, -OH), 8.31 (d, *J* = 8.4 Hz, 2H, -ArH), 8.12 (d, *J* = 8.4 Hz, 2H, -ArH),7.35 (d, *J* = 2.0 Hz, 1H, -ArH), 7.31 (dd, *J* = 8.4, 2.0 Hz, 1H, -ArH), 6.86 (d, *J* = 8.4 Hz, 1H, -ArH), 6.53 (d, *J* = 2.0 Hz, 1H, -ArH), 6.28 (d, *J* = 2.0 Hz, 1H, -ArH); ESI-MS (*m/z*) 432.1 [M+H]^+^, 885.1 [2M+Na]^+^.

*2-(3,4-Dihydroxyphenyl)-5,7-dihydroxy-4-oxo-4H-chromen-3-yl 4-chlorobenzoate* (**4o**). Purified by preparative HPLC. Yield 40%. m.p. 194–195 °C; ^1^H-NMR (400 MHz, *d_6_*-DMSO) δ 8.14 (d, *J* = 8.0 Hz, 2H, -ArH), 7.57 (d, *J* = 8.0 Hz, 2H, -ArH),7.34 (brs, 1H, -ArH), 7.28 (d, *J* = 8.0 Hz, 1H, -ArH), 6.81 (d, *J* = 8.0 Hz, 1H, -ArH), 6.45 (brs, 1H, -ArH), 6.24 (brs, 1H, -ArH); ESI-MS (*m/z*) 440.9 [M+H]^+^.

### 3.9. General Procedure for the Preparation of **11a**–**11d**

To a mixture of compound **9** (200 mg, 0.35 mmol) and potassium carbonate (73 mg, 0.53 mmol) in dry DMF (20 mL), the appropriate halide (0.53 mmol) in dry DMF (2 mL) was add, and the reaction mixture was stirred at 40 °C for 2 h. The mixture was then poured into water (100 mL) and filtered to give the crude product **11a**–**11o** used or the next step directly without further purification.

### 3.10. General Procedure for the Preparation of **12a**–**12d**

10% Palladium on carbon (20 mg) was added to the crude product **11a**–**11o** in a solution of ethanol and 1,4-dioxane (20 mL, 3:1), then the mixture was stirred under hydrogen atmosphere at room temperature for 4 h. The resulting mixture was filtered, washed with EtOH and purified by silica gel column chromatography (CH_2_Cl_2_/MeOH, 40/1, v/v) to give the desired compound **12a**–**12d**.

*2-(3,4-Dihydroxyphenyl)-5,7-dihydroxy-3-methoxy-4H-chromen-4-one* (**12a**). Yield 65%. m.p. 270–271 °C; ^1^H-NMR (DMSO-*d_6_*) δ 12.71 (s, 1H, -OH), 10.85 (brs, 1H, -OH), 9.78 (brs, 1H, -OH), 9.41 (brs, 1H, -OH), 7.55 (d, *J* = 2.0 Hz, 1H, -ArH), 7.45 (dd, *J* = 8.4, 2.0 Hz, 1H, -ArH), 6.90 (d, *J* = 8.4 Hz, 1H, -ArH), 6.41 (d, *J* = 2.0 Hz, 1H, -ArH), 6.19 (d, *J* = 2.0 Hz, 1H, -ArH), 3.78 (s, 3H, -CH_3_); ESI-MS (*m/z*) 317.0 [M+H]^+^.

*2-(3,4-Dihydroxyphenyl)-5,7-dihydroxy-3-isopropoxy-4H-chromen-4-one* (**12b**). Yield 70%. m.p. 259–262 °C; ^1^H-NMR (DMSO-*d_6_*) δ 12.79 (s, 1H, -OH), 10.82 (brs, 1H, -OH), 9.71 (brs, 1H, -OH), 9.36 (brs, 1H, -OH), 7.61 (d, *J* = 2.0 Hz, 1H, -ArH), 7.49 (dd, *J* = 8.4, 2.0 Hz, 1H, -ArH), 6.88 (d, *J* = 8.4 Hz, 1H, -ArH), 6.40 (d, *J* = 2.0 Hz, 1H, -ArH), 6.19 (d, *J* = 2.0 Hz, 1H, -ArH), 4.51(m, 1H, -CH), 1.15 (s, 3H, -CH_3_), 1.14 (s, 3H, -CH_3_); ^13^C-NMR (DMSO-*d_6_*) 178.82, 164.48, 161.77, 156.81, 148.90, 145.37, 135.77, 121.79, 121.44, 116.32, 115.88, 104.53, 98.92, 93.94, 74.74, 22.55; ESI-MS (*m/z*) 345.1 [M+H]^+^, 711.2 [2M+Na]^+^.

*2-(3,4-Dihydroxyphenyl)-5,7-dihydroxy-3-(2-hydroxyethoxy)-4H-chromen-4-one* (**12c**). Yield 61%. m.p. 193–194 °C; ^1^H-NMR (DMSO-*d_6_*) δ 12.70 (s, 1H, -OH), 10.85 (s, 1H, -OH), 9.77 (s, 1H, -OH), 9.33 (s, 1H, -OH), 7.60–7.57 (m, 2H, -ArH), 6.88 (d, *J* = 8.0 Hz, 1H, -ArH), 6.41 (d, *J* = 2.0 Hz, 1H, -ArH), 6.19 (d, *J* = 2.0 Hz, 1H, -ArH), 4.00 (t, *J* = 5.2 Hz, 2H, -CH_2_), 3.66 (brs, 2H, -CH_2_); ESI-MS (*m/z*) 347.2 [M+H]^+^, 715.2 [2M+Na]^+^.

*4-(2-(3,4-Dihydroxyphenyl)-5,7-dihydroxy-4-oxo-4H-chromen-3-yloxy)butanenitrile* (**12d**). Yield 64%. m.p. 169–171 °C; ^1^H-NMR (DMSO­-*d_6_*) δ 12.68 (s, 1H, -OH), 10.86 (brs, 1H, -OH), 9.78 (brs, 1H, -OH), 9.42 (brs, 1H, -OH), 7.50 (d, *J* = 2.0 Hz, 1H, -ArH), 7.42 (dd, *J* = 8.4, 2.0 Hz, 1H, -ArH), 6.91 (d, *J* = 8.4 Hz, 1H, -ArH), 6.41 (d, *J* = 2.0 Hz, 1H, -ArH), 6.20 (d, *J* = 2.0 Hz, 1H, -ArH), 3.97 (t, *J* = 6.0 Hz, 2H, -CH_2_), 2.63–2.60 (m, 2H, -CH_2_); 1.95–1.91 (m, 2H, -CH_2_); ESI-MS (*m/z*) 370.1 [M+H]^+^, 761.2 [2M+Na]^+^.

### 3.11. Biological Assays

#### 3.11.1. Cells and Viruses Preparations

The biological assays were performed following a previously reported procedure [23]. The Huh7.5.1 cell line and the HCV infectious virus J399EM were kindly provided by Xin-Wen Chen, Wuhan Institute of Virology, Chinese Academy of Sciences. Huh7.5.1 cells were cultured in Dulbecco’s modified Eagle’s medium (DMEM) containing 10% (v/v) fetal bovine serum, 0.5 mg/mL G418, 100 IU/mL penicillin, and 100 mg/mL streptomycin.

J399EM is an infectious HCV virus derived from the JFH1 virus (genotype 2a) by insertion of enhanced green fluorescent protein (EGFP) into the HCV NS5A region. The establishment of the infectious J399EM HCV virus has been described previously [24]. J399EM was propagated in Huh7.5.1 cells. Briefly, Huh7.5.1 cells were infected with J399EM at MOI = 0.1 and cultured for 4 days. Culture supernatant was collected and filtered through a 0.45 μm membrane and stored at −80 °C as virus stock. Virus titer was tested in Huh7.5.1 cells. Stocks were serially diluted, and the foci forming units (FFU) were determined as the number of fluorescent colonies formed in infected Huh7.5.1.

#### 3.11.2. Antiviral Assays in Infected Target Cells

All the compounds were first dissolved in DMSO at 50 mM, and then diluted to various concentrations (50 μM, 25 μM, 12.5 μM, 6.3 μM and 3.1 μM). Approximately 1 × 10^4^ Huh7.5.1 cells per well were plated in an opaque 96-well tissue culture plate (Costar 3904). The next day, the medium was replaced by J399EM virus at an MOI of 0.3. Four hours later, the virus inoculum was removed and medium containing different concentrations of compounds was added. After an additional 72 h of incubation, the culture medium was removed and the EGFP autofluorescence was measured by using a luminometer with excitation at 488 nm and emission at 516 nm. Compounds and controls were performed in duplicate and each experiment repeated independently at least twice.

#### 3.11.3. Cell Viability Assays

After the EGFP signal was read, cell viability was measured in the same plate by the MTT [3-(4,5-dimethylthiazol-2-yl)-2,5-diphenyltetrazolium bromide; Sigma-Aldrich, St. Louis, MO, USA] method. Briefly, MTT (1 mg/mL) was dissolved in PBS. Cell culture supernatants were removed and replaced with MTT and further cultured for 4 h; cells were then lysed with 10% sodium dodecyl sulfate (SDS), 50% *N,N*-dimethylformamide, pH 7.2. OD_570_ values were read. The percentage of cell death was calculated against the control well without compounds.

### 3.12. Modeling and Docking

Docking studies were carried out on quercetin and relative substituents using Schrodinger software package [25]. The high-resolution (1.85 Å) X-ray co-crystal structure of HCV NS5B in complex with Mn^2+^ and UTP (PDB code: 1GX6 [22]) was downloaded from the Protein Data Bank. Water molecules were deleted except for those involved in coordination with the two divalent cations in the active site. The crystallographic Mn^2+^ ions were replaced with Mg^2+^ ions. All missing hydrogen atoms were added by standard protein preparation protocol within Maestro followed by energy minimization using OPLS 2005 force field. Restrained minimization was later performed on heavy atoms of the protein with root-mean-square deviation (RMSD) converged to 0.3 Å. The structures of lead compound quercetin and the most active compound of corresponding series (**3i** and **4f**) were built and optimized by Maestro with default settings. Docking of the three compounds were carried out using Glide with standard precision protocol with the Mg^2+^ ions defined as required constraints. The binding conformation with the lowest score was selected to represent the predicted binding mode with HCV NS5B polymerase.

## 4. Conclusions

DKA analogues or isosteres show potent inhibition of HCV NS5B through chelation of the two magnesium ions at the active site. Following our recent report which evidenced the anti-HCV potential of the DKAs mimic quercetin (**2**), efforts were continued in order to delineate the structural features required for the inhibitory effect and improve the anti-HCV potency. Accordingly, two novel quercetin analogues, 7-*O*-arylmethylquercetin derivatives and quercetin-3-*O*-benzoic acid ester derivatives, were synthesized and evaluated for anti-HCV activities. Experimental and modeling results highlighted the importance of introducing an aromatic group at 7-*O* position, which is exemplified by compound **3i**, where a 3-chlorobenzyl substitution at quercetin 7-*O* position confers low micromolar inhibitory activity (EC_50_ = 3.8 μM) and a decent selectivity ratio (SI = 7.2). Moreover, the electronegative carbonyl groups at quercetin 3-*O* position may maintain the coordination with the magnesium ions and more importantly, introduce other interactions with the surrounding residues and thus enhance the anti-HCV activities, which is manifested by compound **4f** (EC_50_ = 9.0 μM and SI > 5.6). Docking studies suggested that the quercetin structures are capable of establishing key coordination with the two magnesium ions as well as interactions with residues at the active site of HCV RdRp. These results demonstrated the feasibility and potential of modifications on quercetin scaffold and provided a promising fundamental to optimize the structures of flavonoids to obtain potent anti-HCV agents.

## Supplementary Materials

Supplementary materials can be accessed at: http://www.mdpi.com/1420-3049/20/04/6978/s1.
