# Supplementary material for: Discovery of Metal Ions Chelator Quercetin Derivatives with Potent Anti-HCV Activities
Source: Molecules. 2015 Apr 16;20(4):6978–99. doi: 10.3390/molecules20046978 (PMC6272327; doi:10.3390/molecules20046978)
Supplement: Supplementary file 1 [file molecules-20-06978-s001.pdf]

# Supplementary Data

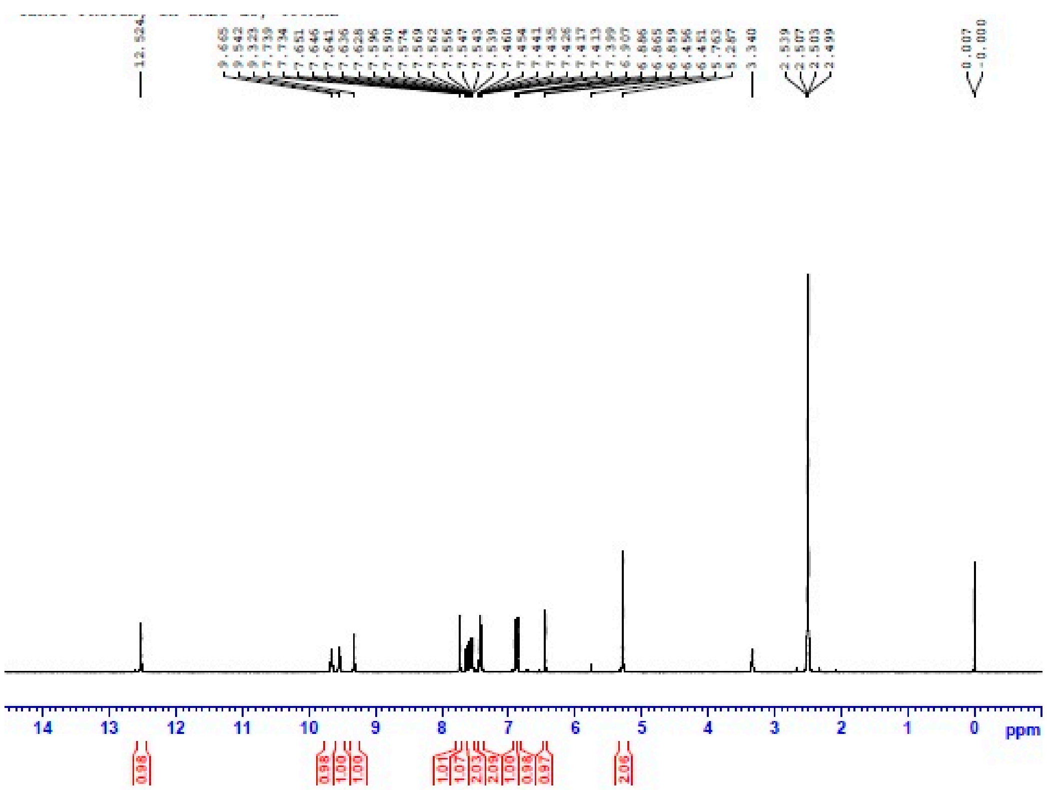

**Figure S1.** $^{1}$H-NMR spectrum of **3c** (DMSO-$d_6$).

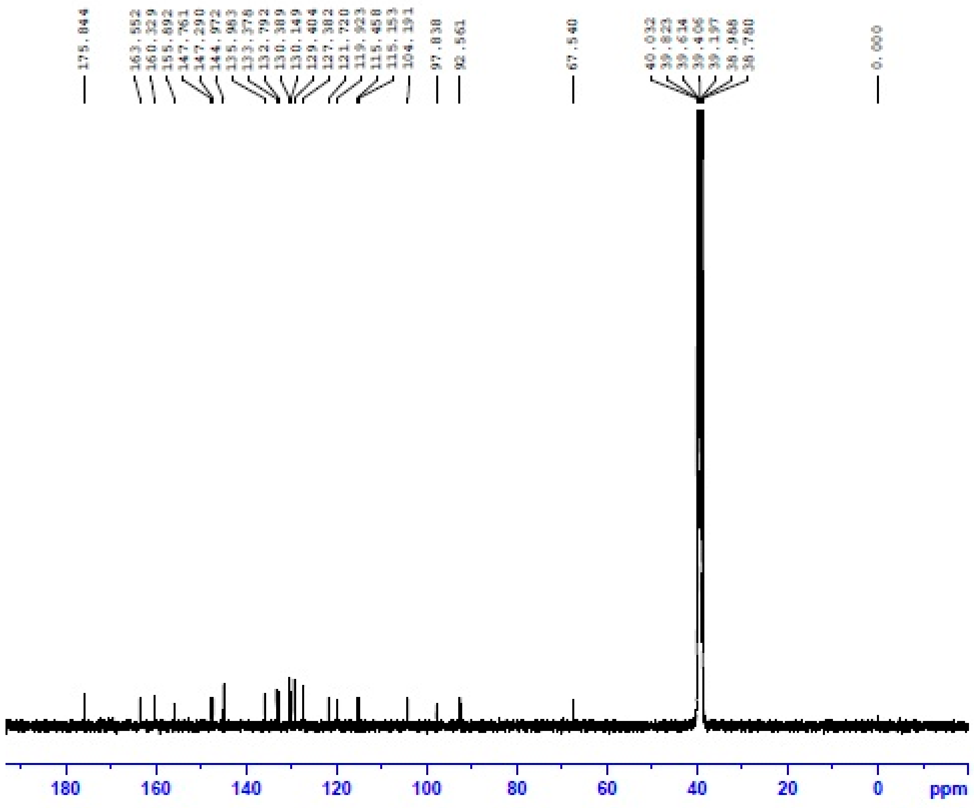

**Figure S2.** $^{13}$C-NMR spectrum of **3c** (DMSO-$d_6$).

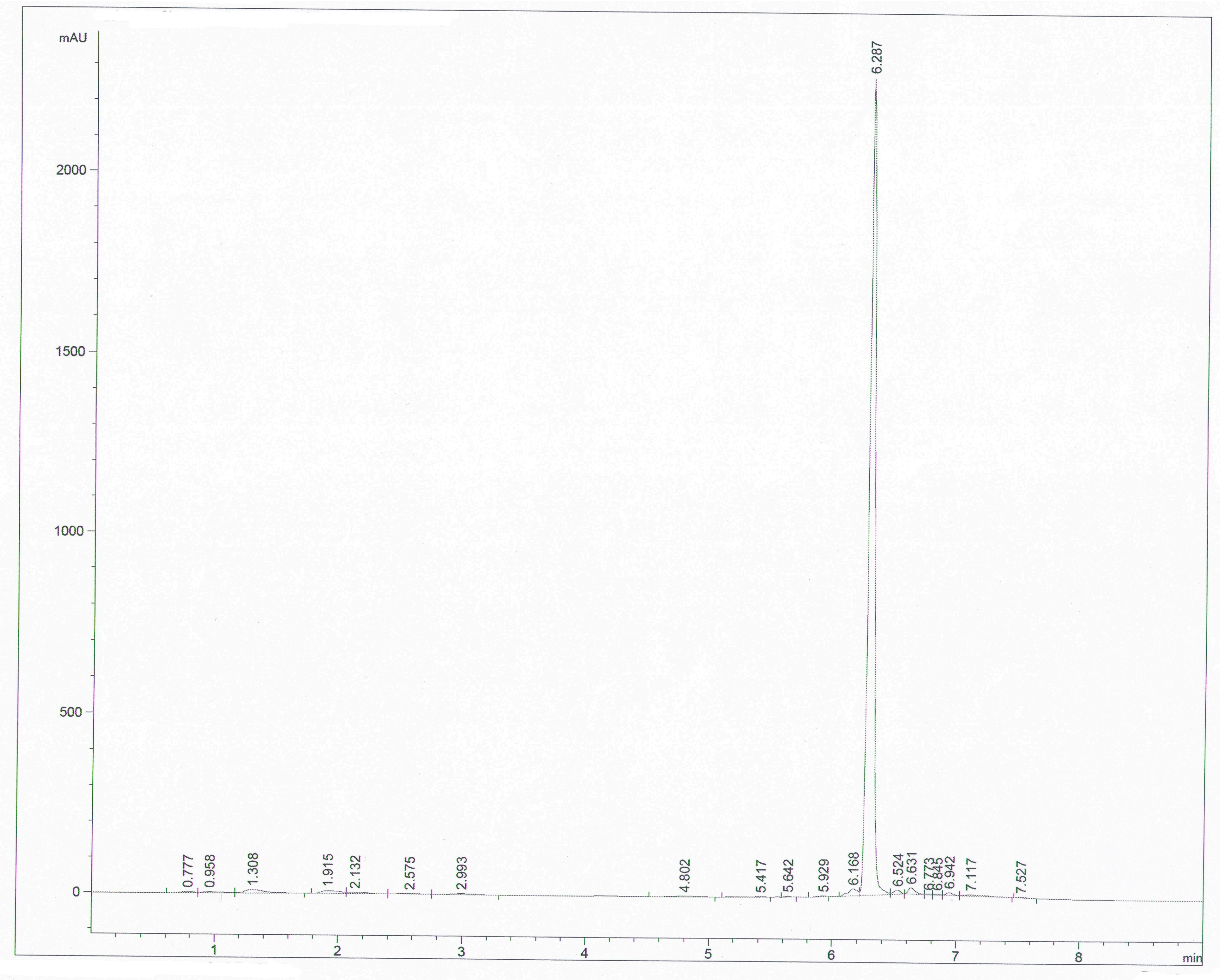


**Figure S3.** HPLC analysis of **3c**. Compound **3c** was analyzed by Agilent Technologies HPLC system and C18 reversed-phase HPLC column. Mobile phase consisted of a solvent mixture of methanol: 0.1% formic acid in the ratio 60:40 (v/v). The flow rate and test wavelength were 0.250 mL/min and 254 nm, respectively.

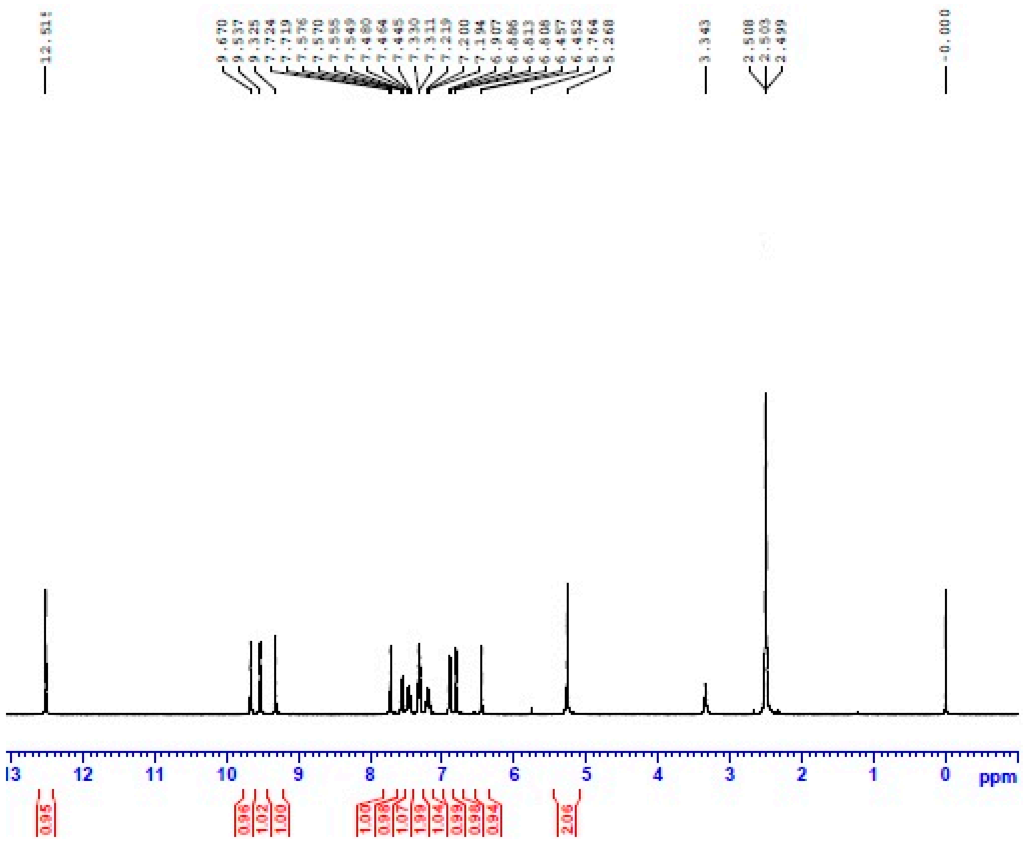

**Figure S4.** $^{1}H$-NMR spectrum of **3h** (DMSO-$d_6$).

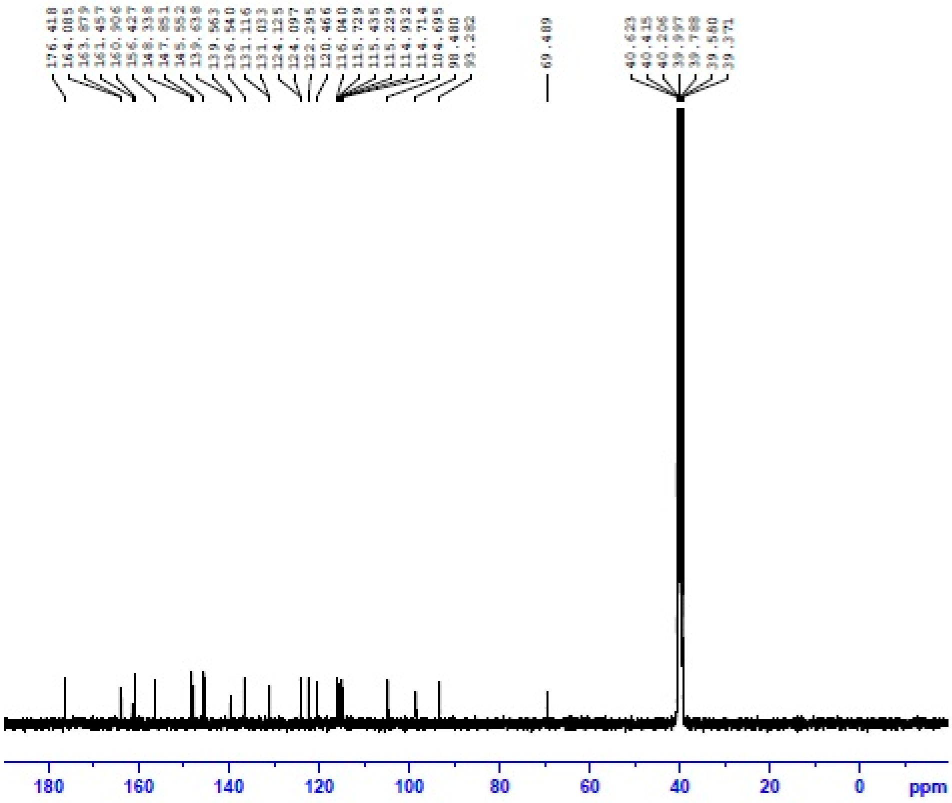

**Figure S5.** $^{13}C$-NMR spectrum of **3h** (DMSO-$d_6$).

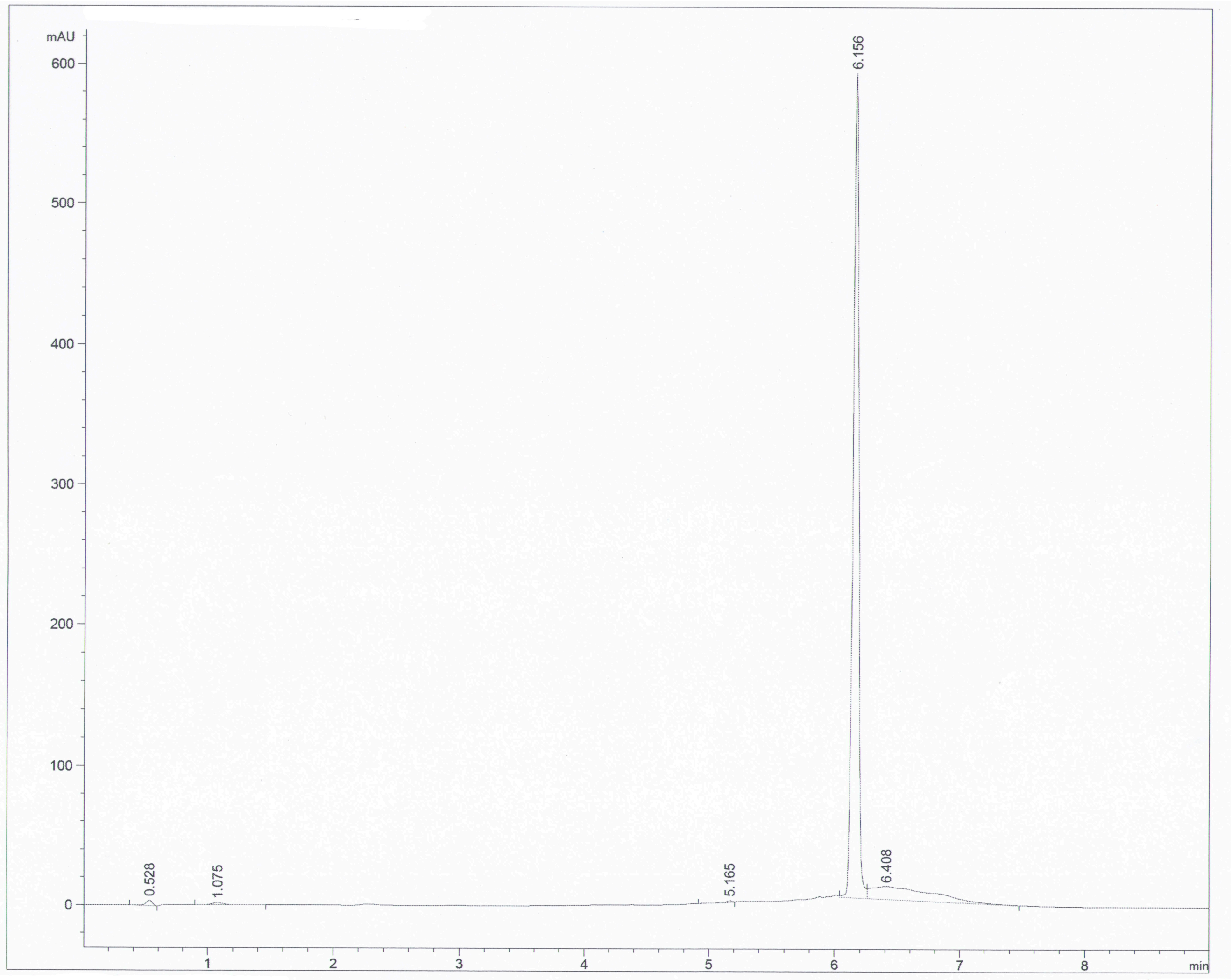


**Figure S6.** HPLC analysis of **3h**. Compound **3h** was analyzed by Agilent Technologies HPLC system and C18 reversed-phase HPLC column. Mobile phase consisted of a solvent mixture of methanol: 0.1% formic acid in the ratio 60:40 (v/v). The flow rate and test wavelength were 0.250 mL/min and 254 nm, respectively.

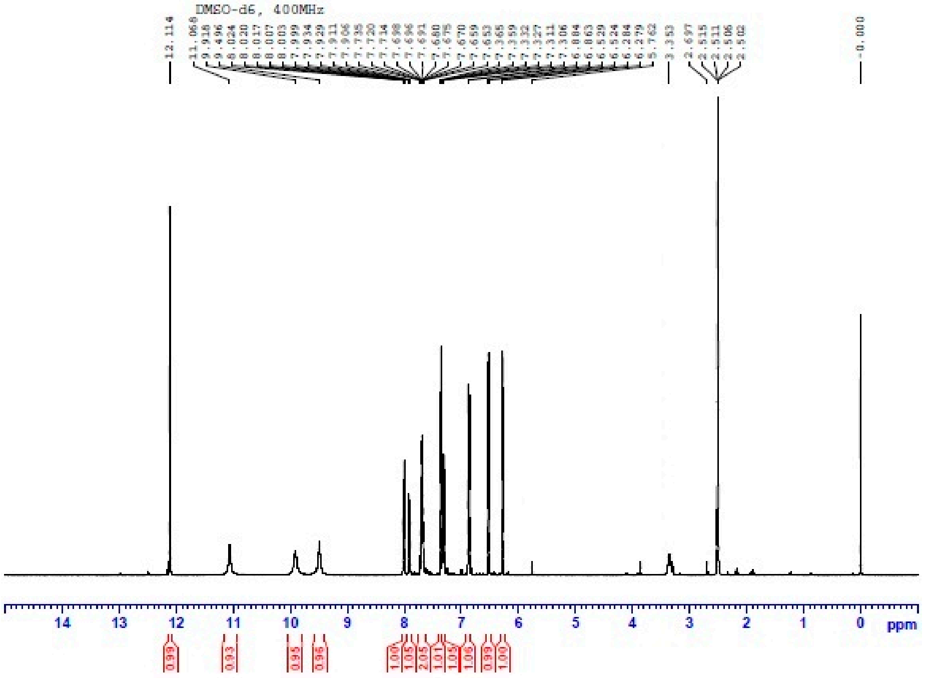


**Figure 7.** $^{1}$H-NMR spectrum of **4f** (DMSO-$d_6$).

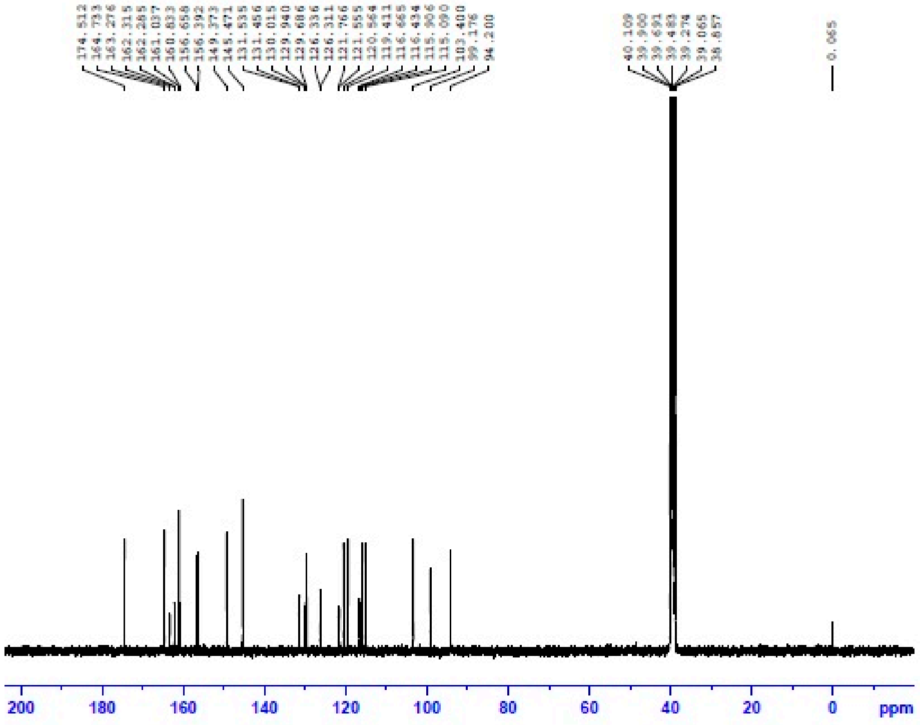


**Figure S8.** $^{13}$C-NMR spectrum of **4f** (DMSO-$d_6$).

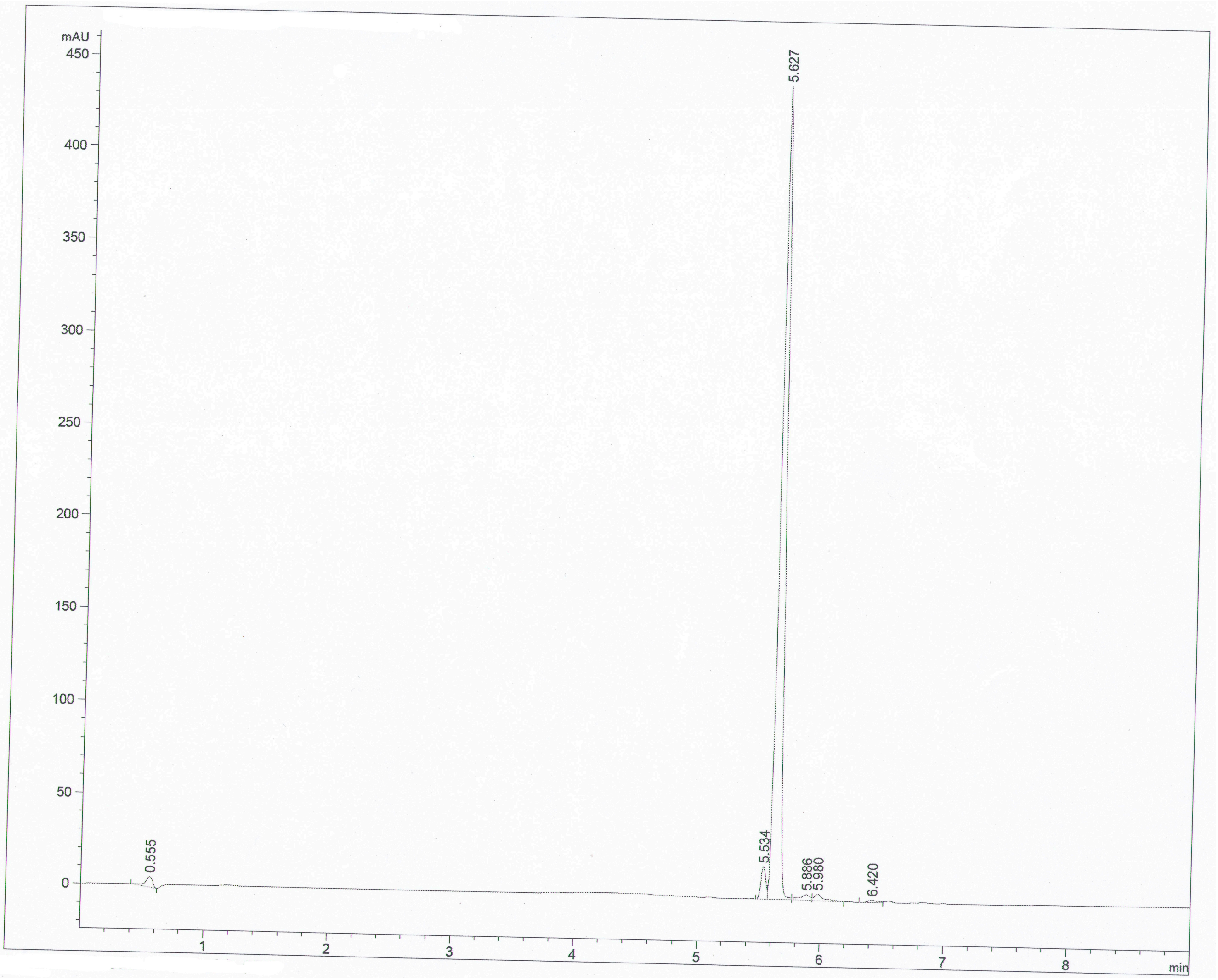


**Figure S9.** HPLC analysis of **4f**. Compound **4f** was analyzed by Agilent Technologies HPLC system and C18 reversed-phase HPLC column. Mobile phase consisted of a solvent mixture of methanol: 0.1% formic acid in the ratio 60:40 (v/v). The flow rate and test wavelength were 0.250 mL/min and 254 nm, respectively.

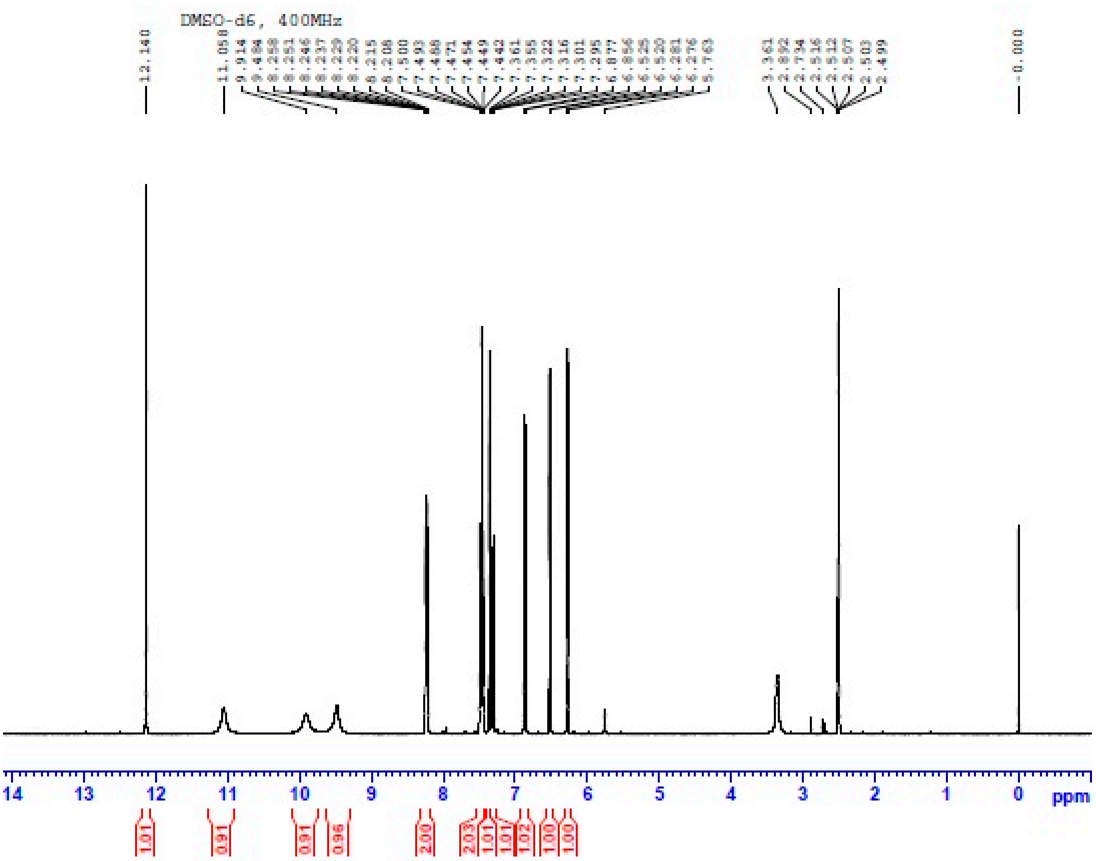


**Figure S10.** $^{1}$H-NMR spectrum of **4k** (DMSO-$d_6$).

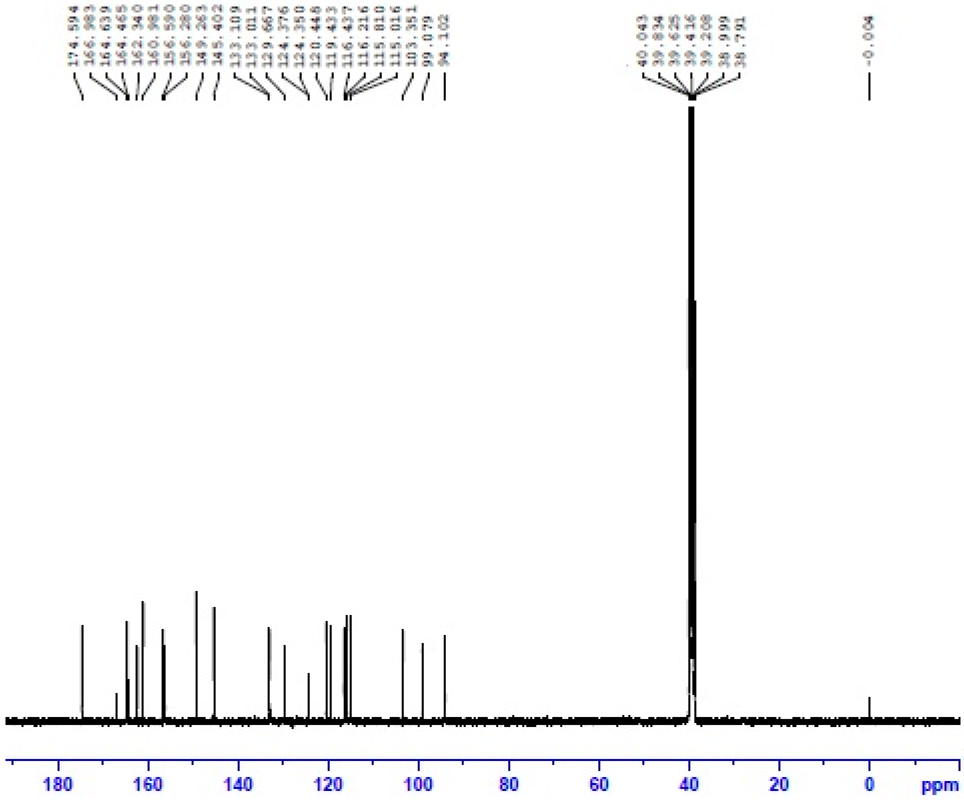


**Figure S11.** $^{13}$C-NMR spectrum of **4k** (DMSO-$d_6$).

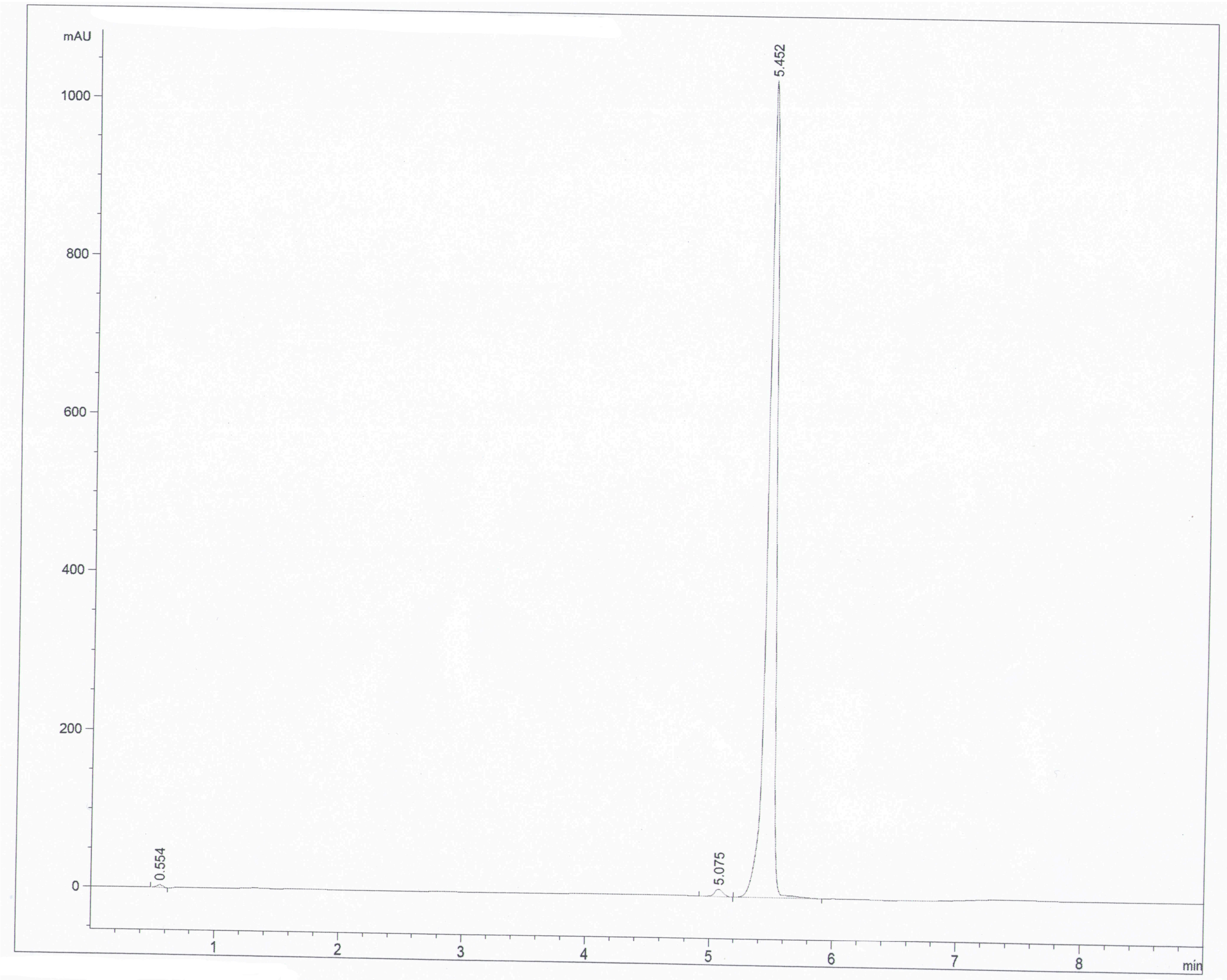


**Figure S12.** HPLC analysis of **4k**. Compound **4k** was analyzed by Agilent Technologies HPLC system and C18 reversed-phase HPLC column. Mobile phase consisted of a solvent mixture of methanol: 0.1% formic acid in the ratio 60:40 (v/v). The flow rate and test wavelength were 0.250 mL/min and 254 nm, respectively.
